# Supplementary material for: Hybrid Models and Biological Model Reduction with PyDSTool
Source: PLoS Comput Biol. 2012 Aug 9;8(8):e1002628. doi: 10.1371/journal.pcbi.1002628 (PMC3415397; doi:10.1371/journal.pcbi.1002628)
Supplement: Text S4 — Complete source code for the PyDSTool package (version 0.88.120504). Includes API documentation and help files linking to web pages. This file is identical to the current public release on Sourceforge.net. (ZIP) [file pcbi.1002628.s004.zip › PyDSTool/html/PyDSTool.Model-pysrc.html]

xml version="1.0" encoding="ascii"?


PyDSTool.Model


| Home | Trees | Indices | Help | PyDSTool |
| --- | --- | --- | --- | --- |

Package PyDSTool ::
Module Model

[hide private]

[frames] | no frames]

# Source Code for Module PyDSTool.Model

```
   1  """General purpose (hybrid) model class, and associated hybrid trajectory
 
   2  and variable classes.
 
   3  
 
   4     Robert Clewley, March 2005.
 
   5  
 
   6  A Model object's hybrid trajectory can be treated as a curve, or as
 
   7  a mapping. Call the model object with the trajectory name, time(s), and
 
   8  set the 'asmap' argument to be True to use an integer time to select the
 
   9  trajectory segment. These are numbered from zero.
 
  10  
 
  11  A trajectory value in a Model object's 'trajectories' dictionary
 
  12  attribute is a HybridTrajectory object, having the following
 
  13  attributes (among others):
 
  14  
 
  15      timeInterval is the entire time interval for the trajectory.
 
  16  
 
  17      timePartitions is a sequence of time_intervals (for each trajectory
 
  18          segment in trajseq), and
 
  19  
 
  20      trajSeq is a list of epoch or regime trajectory segments [traj_0, traj_1,
 
  21          ..., traj_(R-1)],
 
  22  
 
  23          where traj_i is a callable Trajectory or HybridTrajectory object.
 
  24  
 
  25      eventStruct is the event structure used to determine that trajectory.
 
  26  
 
  27      events is a dictionary of event names -> list of times at which that
 
  28        event took place.
 
  29  
 
  30      modelNames is a list of the generators used for the trajectory (one per
 
  31                                                               partition).
 
  32  
 
  33      variables is a dictionary that mimics the variables of the trajectory.
 
  34  """ 
  35  
 
  36  # ----------------------------------------------------------------------------
 
  37  
 
  38  ## PyDSTool imports
 
  39  import Generator, Events, MProject 
  40  from utils import * 
  41  from common import * 
  42  from errors import * 
  43  from Interval import * 
  44  from Trajectory import * 
  45  from Variable import * 
  46  from Points import * 
  47  from ModelSpec import * 
  48  from Symbolic import isMultiRef 
  49  from parseUtils import isHierarchicalName, NAMESEP, mapNames, symbolMapClass 
  50  
 
  51  ## Other imports
 
  52  import math, sys 
  53  from numpy import Inf, NaN, isfinite, sign, abs, array, arange, \
 
  54       zeros, concatenate, transpose, shape 
  55  from numpy import sometrue, alltrue, any, all 
  56  import copy 
  57  from time import clock 
  58  import pprint 
  59  
 
  60  __all__ = ['Model', 'HybridModel', 'NonHybridModel',
 
  61             'boundary_containment', 'boundary_containment_by_postproc',
 
  62             'boundary_containment_by_event',
 
  63             'domain_test'] 
  64  
 
  65  # ----------------------------------------------------------------------------
 
  66  
 
  67  
 


68 -class boundary_containment(MProject.qt_feature_leaf):


69      # not implemented using metrics because the metrics are trivial
 
  70      # and cause a lot of overhead for this often-evaluated feature
 


71 -    def __init__(self, name, description='', pars=None):


72          MProject.qt_feature_leaf.__init__(self, name, description, pars) 
  73          try: 
  74              pars.thresh 
  75          except AttributeError: 
  76              raise ValueError("Missing threshold specification") 
  77          try: 
  78              # tolerance for small rounding errors
 
  79              pars.abseps 
  80          except AttributeError: 
  81              self.pars.abseps = 0 
  82          try: 
  83              assert pars.interior_dirn in [-1, 0, 1] 
  84          except AttributeError: 
  85              raise ValueError("Missing interior direction specification") 
  86          except AssertionError: 
  87              raise ValueError("Invalid interior direction specification value "
 
  88                  " use -1 for 'below', 1 for 'above', or 0 for 'discrete domain'") 
  89          try: 
  90              self.pars.coordname 
  91          except AttributeError: 
  92              # test all coords at once
 
  93              self.pars.coordname = None 
  94          else: 
  95              assert isinstance(self.pars.coordname, str), \
 
  96                     "Coordinate name must be a string" 
  97  
 
  98          def evaluate(self, target): 
  99              raise NotImplementedError("Only call this method on a concrete "
 
 100                      "sub-class")

 101  
 
 102  
 


103 -class boundary_containment_by_event(boundary_containment):


104 -    def __init__(self, name, description='', pars=None):


105          boundary_containment.__init__(self, name, description, pars) 
 106          try: 
 107              self.pars.bd_eventname 
 108          except AttributeError: 
 109              raise ValueError("Missing boundary event name") 
 110          # assume that the supplied model will correspond to the source of
 
 111          # trajectories in evaluate method
 
 112          try: 
 113              self.pars.model 
 114          except AttributeError: 
 115              raise ValueError("Missing model associated with event")

 116          # have not verified that event is present in model
 
 117  
 


118 -    def evaluate(self, traj):


119          # verify whether event exists and was flagged in associated model
 
 120          try: 
 121              evpts = traj.getEvents(self.pars.bd_eventname) 
 122          except ValueError, errinfo: 
 123              print errinfo 
 124              raise RuntimeError("Could not find flagged events for this trajectory") 
 125          try: 
 126              evpt = evpts[self.pars.coordname] 
 127          except KeyError: 
 128              raise ValueError("No such coordinate %s in the defined event"%self.pars.coordname) 
 129          except TypeError: 
 130              # no events of this kind were found, so passed feature eval test
 
 131              # dereferencing None (unsubscriptable object)
 
 132              if self.pars.abseps > 0: 
 133                  # would like to re-evaluate event at its threshold+abseps, but
 
 134                  # leave for now
 
 135                  print "Warning -- Boundary containment feature %s:"%self.name 
 136                  print " Check for uncertain case using events not implemented" 
 137                  self.results.output = None 
 138                  self.results.uncertain = False 
 139              else: 
 140                  self.results.output = None 
 141                  self.results.uncertain = False 
 142              return True 
 143          else: 
 144              # event found
 
 145              if self.pars.abseps > 0: 
 146                  # would like to re-evaluate event at its threshold+abseps, but
 
 147                  # leave for now
 
 148                  print "Warning -- Boundary containment feature %s:"%self.name 
 149                  print " Check for uncertain case using events not implemented" 
 150                  self.results.output = evpt[0]  # only use first event (in case not Terminal) 
 151                  self.results.uncertain = False 
 152              else: 
 153                  self.results.output = evpt[0]  # only use first event (in case not Terminal) 
 154                  self.results.uncertain = False 
 155              return False

 156  
 


157 -    def _find_idx(self):


158          """Helper function for finding index in trajectory meshpoints
 
 159          at which containment first failed.""" 
 160          if self.results.satisfied: 
 161              # Trajectory satisfied constraint!
 
 162              return None 
 163          return len(self.results.output)

 164  
 
 165  
 


166 -class boundary_containment_by_postproc(boundary_containment):


167 -    def evaluate(self, traj):


168          diffs = [p - self.pars.thresh for p in \
 
 169                   traj.sample(coords=self.pars.coordname)] 
 170          if self.pars.verbose_level > 1: 
 171              print "%s diffs in coord %s ="%(self.name,self.pars.coordname), diffs 
 172          res_strict = array([sign(d) \
 
 173                       == self.pars.interior_dirn for d in diffs]) 
 174          satisfied_strict = alltrue(res_strict) 
 175          if self.pars.abseps > 0: 
 176              if self.pars.interior_dirn == 0: 
 177                  # especially for discrete domains
 
 178                  res_loose = array([abs(d) < self.pars.abseps for d in diffs]) 
 179              else: 
 180                  res_loose = array([sign(d + self.pars.interior_dirn*self.pars.abseps) \
 
 181                                     == self.pars.interior_dirn for d in diffs]) 
 182              satisfied_loose = alltrue(res_loose) 
 183              self.results.output = res_loose 
 184              # if p values are *outside* thresh by up to abseps amount
 
 185              # then flag this as 'uncertain' for use by domain_test class's
 
 186              # transversality testing
 
 187              self.results.uncertain = satisfied_loose and not satisfied_strict 
 188              return satisfied_loose 
 189          else: 
 190              self.results.output = res_strict 
 191              self.results.uncertain = sometrue(array(diffs)==0) 
 192              return alltrue(res_strict)

 193  
 


194 -    def _find_idx(self):


195          """Helper function for finding index in trajectory meshpoints
 
 196          at which containment first failed""" 
 197          if self.results.satisfied: 
 198              # Trajectory satisfied constraint!
 
 199              return None 
 200          res = self.results.output 
 201          if res[0] == -1: 
 202              adjusted_res = list((res + 1) != 0) 
 203          elif res[0] == 1: 
 204              adjusted_res = list((res - 1) != 0) 
 205          else: 
 206              # starts with 0 already
 
 207              adjusted_res = list(res != 0) 
 208          # find first index at which value is non-zero
 
 209          # should never raise ValueError because this method is
 
 210          # only run if there was a sign change found
 
 211          return adjusted_res.index(True)

 212  
 
 213  
 


214 -class domain_test(MProject.qt_feature_node):


215 -    def __init__(self, name, description='', pars=None):


216          MProject.qt_feature_node.__init__(self, name, description, pars) 
 217          try: 
 218              self.pars.interval 
 219          except AttributeError: 
 220              raise ValueError("Missing domain interval specification") 
 221          try: 
 222              # tolerance for small rounding errors
 
 223              self.pars.abseps 
 224          except AttributeError: 
 225              if isinstance(self.pars.interval, Interval): 
 226                  # Interval type passed, inherit its abseps
 
 227                  self.pars.abseps = self.pars.interval._abseps 
 228              else: 
 229                  self.pars.abseps = 0 
 230                  if not isinstance(self.pars.interval, (tuple, list)): 
 231                      # assume a singleton numeric type passed
 
 232                      self.pars.interval = [self.pars.interval, self.pars.interval] 
 233                  elif len(self.pars.interval)==1: 
 234                      # singleton passed, so copy the value for both
 
 235                      # "endpoints" so that xlo_bc etc. below will work
 
 236                      self.pars.interval = [self.pars.interval[0],
 
 237                                            self.pars.interval[0]] 
 238          self.isdiscrete = self.pars.interval[0] == self.pars.interval[1] 
 239          try: 
 240              self.pars.coordname 
 241          except AttributeError: 
 242              raise ValueError("Missing coordinate name") 
 243          try: 
 244              self.pars.derivname 
 245          except AttributeError: 
 246              raise ValueError("Missing coordinate derivative name") 
 247  
 
 248          # multiply interior directions by the integer value of not self.isdiscrete
 
 249          # in order to set them to zero when the "interval" is actually a singleton
 
 250          # value for a discrete domain. That fixes the boundary containment evaluation
 
 251          # code which compares the sign of coord differences with that interior
 
 252          # direction value.
 
 253          xlo_bc = boundary_containment_by_postproc('x_test_lo',
 
 254                               description='Test x lower bound',
 
 255                               pars=args(thresh=self.pars.interval[0],
 
 256                                         interior_dirn=1*int(not self.isdiscrete),
 
 257                                         abseps=self.pars.abseps,
 
 258                                         coordname=self.pars.coordname)) 
 259          xhi_bc = boundary_containment_by_postproc('x_test_hi',
 
 260                               description='Test x upper bound',
 
 261                               pars=args(thresh=self.pars.interval[1],
 
 262                                         interior_dirn=-1*int(not self.isdiscrete),
 
 263                                         abseps=self.pars.abseps,
 
 264                                         coordname=self.pars.coordname)) 
 265          dxlo_bc = boundary_containment_by_postproc('dx_test_lo',
 
 266                               description='Test dx at lower x bound',
 
 267                               pars=args(thresh=0,
 
 268                                         interior_dirn=1*int(not self.isdiscrete),
 
 269                                         abseps=0,
 
 270                                         coordname=self.pars.derivname)) 
 271          dxhi_bc = boundary_containment_by_postproc('dx_test_hi',
 
 272                               description='Test dx at upper x bound',
 
 273                               pars=args(thresh=0,
 
 274                                         interior_dirn=-1*int(not self.isdiscrete),
 
 275                                         abseps=0,
 
 276                                         coordname=self.pars.derivname)) 
 277          self.subfeatures = {'x_test_lo': xlo_bc,
 
 278                              'dx_test_lo': dxlo_bc,
 
 279                              'x_test_hi': xhi_bc,
 
 280                              'dx_test_hi': dxhi_bc}

 281  
 


282 -    def evaluate(self, traj):


283          # Arg is a traj!
 
 284          for sf in self.subfeatures.values(): 
 285              self.propagate_verbosity(sf) 
 286              sf.super_pars = self.pars 
 287              sf.super_results = self.results 
 288          xlo_bc = self.subfeatures['x_test_lo'] 
 289          xlo_test = xlo_bc(traj) 
 290          if xlo_bc.results.uncertain: 
 291              if self.pars.verbose_level > 0: 
 292                  print "Lo bd uncertain" 
 293              if self.isdiscrete: 
 294                  # accept uncertain case for discrete domain
 
 295                  xlo_test = True 
 296              else: 
 297                  # check transversality at critical (boundary) value of domain
 
 298                  xlo_test = self.subfeatures['dx_test_lo'](traj) 
 299          xhi_bc = self.subfeatures['x_test_hi'] 
 300          xhi_test = xhi_bc(traj) 
 301          if xhi_bc.results.uncertain: 
 302              if self.pars.verbose_level > 0: 
 303                  print "Hi bd uncertain" 
 304              if self.isdiscrete: 
 305                  # accept uncertain case for discrete domain
 
 306                  xhi_test = True 
 307              else: 
 308                  # check transversality at critical (boundary) value of domain
 
 309                  xhi_test = self.subfeatures['dx_test_hi'](traj) 
 310          for sf in self.subfeatures.values(): 
 311              self.results[sf.name] = sf.results 
 312          return xlo_test and xhi_test

 313  
 


314 -    def _find_idx(self):


315          """Helper function for finding lowest index in trajectory meshpoints
 
 316          at which domain test first failed""" 
 317          if self.results.satisfied: 
 318              # Trajectory satified domain conditions!
 
 319              return None 
 320          lowest_idx = Inf 
 321          for sfname, sf in self.subfeatures.items(): 
 322              if self.pars.verbose_level > 0: 
 323                  print "\n", sfname, sf.results 
 324              try: 
 325                  res = list(self.results[sfname].output) 
 326              except AttributeError: 
 327                  # dxdt transversality test was not run so ignore
 
 328                  continue 
 329              if sf.results.satisfied: 
 330                  continue 
 331              if self.pars.verbose_level > 0: 
 332                  print res 
 333              # Find first index at which value is non-zero.
 
 334              # Will not raise ValueError because test satisfaction
 
 335              # was already checked, so must have a zero crossing
 
 336              idx = res.index(False) 
 337              if idx < lowest_idx: 
 338                  lowest_idx = idx 
 339          return lowest_idx

 340  
 
 341  
 
 342  # -------------------
 
 343  
 
 344  
 


345 -class Model(object):


346      """
 
 347      General-purpose Hybrid and Non-Hybrid Model abstract class.
 
 348  
 
 349      """ 
 350      _needKeys = ['name', 'modelInfo'] 
 351      _optionalKeys = ['ics', 'mspecdict', 'verboselevel',
 
 352                      'norm', 'tdata', 'eventPars', 'abseps'] 
 353      # query keys for 'query' method
 
 354      _querykeys = ['pars', 'parameters', 'events', 'submodels',
 
 355                    'ics', 'initialconditions', 'vars', 'variables',
 
 356                    'auxvariables', 'auxvars', 'vardomains', 'pardomains',
 
 357                    'abseps'] 
 358      # valid keys for 'set' method
 
 359      _setkeys = ['pars', 'algparams', 'checklevel', 'abseps',
 
 360                  'ics', 'inputs', 'tdata', 'restrictDSlist',
 
 361                  'globalt0', 'verboselevel', 'inputs_t0'] 
 362  
 


363 -    def __init__(self, legit, *a, **kw):


364          # legit is a way to ensure that instances of this abstract class
 
 365          # are not created directly
 
 366          if not legit==True: 
 367              # use explicit comparison to True otherwise kw argument will
 
 368              # eval to True, which is not what we want
 
 369              raise RuntimeError("Only use HybridModel or NonHybridModel classes") 
 370          if len(a) > 0: 
 371              if len(a) == 1 and isinstance(a[0], dict): 
 372                  if intersect(a[0].keys(),kw.keys()) != []: 
 373                      raise ValueError("Cannot have initialization keys "
 
 374                          "common to both dictionary and keyword arguments") 
 375                  kw.update(a[0]) 
 376              else: 
 377                  raise ValueError("Non-keyword arguments must be a single "
 
 378                                     "dictionary") 
 379          try: 
 380              self.name = kw['name'] 
 381              # modelInfo is a dict mapping model names --> a dict of:
 
 382              #   'dsi': GeneratorInterface or ModelInterface object
 
 383              #   'swRules': dict of switching rules (transitions between
 
 384              #      trajectory segments)
 
 385              #   'globalConRules': list of global consistency DS names
 
 386              #   'domainTests': dictionary of variable name -> domain
 
 387              self.modelInfo = kw['modelInfo'] 
 388          except KeyError: 
 389              raise KeyError('Necessary keys missing from argument') 
 390          _foundKeys = len(self._needKeys) 
 391  
 
 392          # by default, 'observables' are all variables that are common
 
 393          # to the Model/Generator objects in self.modelInfo, and the
 
 394          # 'internals' are those remaining generate obsvars, intvars...
 
 395          # aux vars are those aux vars common to *all* sub-models.
 
 396          # create self.obsvars, self.intvars, self.auxvars
 
 397          self.defaultVars() 
 398  
 
 399          # trajectories is a dict of trajectory segments (in sequence)
 
 400          # ... can only add one at a time!
 
 401          self.trajectories = {} 
 402          self.trajectory_defining_args = {} 
 403          # registry of generators or models (depending on sub-class)
 
 404          # Using registry provides a shortcut for accessing a sub-model regardless
 
 405          # of whether it's a Generator or a Model class
 
 406          self.registry = {} 
 407          for name, infodict in self.modelInfo.iteritems(): 
 408              # set super model tag of ds object (which is either a
 
 409              # ModelInterface or Generator)
 
 410              try: 
 411                  infodict['dsi']._supermodel = self.name 
 412              except KeyError: 
 413                  raise TypeError("Invalid modelInfo entry found with name %s"%name) 
 414              self.registry[name] = infodict['dsi'].model 
 415  
 
 416          self.diagnostics = Diagnostics() 
 417          # set initial conditions if specified already (if not, they must
 
 418          # be specified before or during when compute() is called)
 
 419          self.icdict = {} 
 420          if 'ics' in kw: 
 421              self.icdict = dict(kw['ics']) 
 422              _foundKeys += 1 
 423          else: 
 424              self.icdict = {}.fromkeys(self.allvars, NaN) 
 425          if 'tdata' in kw: 
 426              self.tdata = kw['tdata'] 
 427              _foundKeys += 1 
 428          else: 
 429              self.tdata = None 
 430          if 'norm' in kw: 
 431              self._normord = kw['norm'] 
 432              _foundKeys += 1 
 433          else: 
 434              self._normord = 2 
 435          if 'abseps' in kw: 
 436              # impose this absolute epsilon (small scale) on all components
 
 437              self._abseps = kw['abseps'] 
 438              _foundKeys += 1 
 439          else: 
 440              # All components use their defaults
 
 441              self._abseps = self.query('abseps') 
 442          if 'verboselevel' in kw: 
 443              if kw['verboselevel'] in [0,1,2]: 
 444                  self.verboselevel = kw['verboselevel'] 
 445              else: 
 446                  raise ValueError("Verbosity level value must be 0, 1, or 2") 
 447              _foundKeys += 1 
 448          else: 
 449              self.verboselevel = 0 
 450          if 'mspecdict' in kw: 
 451              self._mspecdict = kw['mspecdict'] 
 452              _foundKeys += 1 
 453          else: 
 454              self._mspecdict = None 
 455          if 'eventPars' in kw: 
 456              self._eventPars = kw['eventPars'] 
 457              _foundKeys += 1 
 458          else: 
 459              self._eventPars = {} 
 460          if _foundKeys < len(kw): 
 461              raise KeyError('Invalid keys found in arguments') 
 462          # If not already created, a True result for
 
 463          # self.haveJacobian() means that the Model will have a
 
 464          # Jacobian method made during compute(), which
 
 465          # references the appropriate _auxfn_Jac function in the Generator
 
 466          # objects.
 
 467          #
 
 468          # Use this dict to record if any external input t0 time shift
 
 469          # values are linked to a parameter value, for automatic
 
 470          # updating before trajectory computation. (Internal use)
 
 471          self._inputt0_par_links = {}

 472  
 
 473  
 


474 -    def __len__(self):


475          """Return number of sub-models""" 
 476          return len(self.registry)

 477  
 


478 -    def sub_models(self):


479          """Return a list of all sub-model instances (model interfaces or generators)""" 
 480          return self.registry.values()

 481  
 


482 -    def _makeDefaultVarNames(self):


483          """Return default observable, internal, and auxiliary variable names
 
 484          from modelInfo.""" 
 485          obsvars = [] 
 486          auxvars = [] 
 487          all_known_varnames = [] 
 488          for infodict in self.modelInfo.values(): 
 489              varnames = infodict['dsi'].query('variables') 
 490              all_known_varnames.extend(varnames) 
 491              auxvarnames = infodict['dsi'].query('auxvariables') 
 492              if auxvars == []: 
 493                  # first ds to have auxvars, so just add them all
 
 494                  auxvars.extend(auxvarnames) 
 495              else: 
 496                  auxvars = intersect(auxvars, auxvarnames) 
 497              if obsvars == []: 
 498                  # first ds, so add them all
 
 499                  obsvars = varnames 
 500              else: 
 501                  obsvars = intersect(obsvars, varnames) 
 502          intvars = remain(all_known_varnames, obsvars) 
 503          return (obsvars, intvars, auxvars)

 504  
 


505 -    def _generateParamInfo(self):


506          """Record parameter info locally, for future queries.
 
 507          Internal use only.
 
 508          """ 
 509          # use query method in case model in registry is a wrapped Generator
 
 510          # that uses _ versions of hierarchical names that are used natively
 
 511          # here.
 
 512          self.pars = {} 
 513          for model in self.registry.values(): 
 514              try: 
 515                  self.pars.update(model.query('pars')) 
 516              except AttributeError: 
 517                  # no pars present
 
 518                  pass

 519  
 


520 -    def showDef(self, target=None, type=''):


521          """type = 'spec', 'auxspec', 'auxfnspec', 'events', or 'modelspec'
 
 522          (leave blank for the first *four* together).
 
 523  
 
 524          'spec', 'auxspec' and 'auxfnspec', 'events' refer to the compiled
 
 525          target language code for the specifications. 'modelspec' refers to
 
 526          the pre-compiled abstract specifications of the model.""" 
 527          if target is None: 
 528              print "Use showInfo() to find names of defined sub-models" 
 529              return 
 530          else: 
 531              showAll = type=='' 
 532              try: 
 533                  if type=='spec' or showAll: 
 534                      self.registry[target].showSpec() 
 535                  if type=='auxspec' or showAll: 
 536                      self.registry[target].showAuxSpec() 
 537                  if type=='auxfnspec' or showAll: 
 538                      self.registry[target].showAuxFnSpec() 
 539                  if type=='events' or showAll: 
 540                      self.registry[target].showEventSpec() 
 541                  if type=='modelspec': 
 542                      if self._mspecdict is None: 
 543                          raise PyDSTool_ExistError("Cannot use this function "
 
 544                              "for models not defined through ModelSpec") 
 545                      info(self._mspecdict[target]['modelspec'].flattenSpec(\
 
 546                                  [self.modelInfo[target]['dsi'].get('indepvariable').name])) 
 547              except KeyError: 
 548                  raise ValueError("Model named %s is not known"%target)

 549  
 


550 -    def showSpec(self):


551          for ds in self.registry.values(): 
 552              ds.showSpec()

 553  
 


554 -    def showAuxSpec(self):


555          for ds in self.registry.values(): 
 556              ds.showAuxSpec()

 557  
 


558 -    def showAuxFnSpec(self):


559          for ds in self.registry.values(): 
 560              ds.showAuxFnSpec()

 561  
 


562 -    def showEventSpec(self):


563          for ds in self.registry.values(): 
 564              ds.showEventSpec()

 565  
 


566 -    def current_defining_args(self):


567          return args(pars=self.pars, ics=self.icdict,
 
 568                          tdata=self.tdata)

 569  
 


570 -    def has_exact_traj(self, trajname, info):


571          """Compare self.pars, self.icdict and self.tdata
 
 572          against what's stored for a previously computed trajectory,
 
 573          so that re-computation can be avoided.
 
 574          """ 
 575          try: 
 576              return info == self.trajectory_defining_args[trajname] 
 577          except KeyError: 
 578              # not even a trajectory of this name
 
 579              return False

 580  
 


581 -    def _prepareCompute(self, trajname, **kw):


582          foundKeys = 0 
 583          if 'verboselevel' in kw: 
 584              self.set(verboselevel=kw['verboselevel']) 
 585              foundKeys += 1 
 586          else: 
 587              self.set(verboselevel=0) 
 588          if 'ics' in kw: 
 589              self.icdict = dict(kw['ics']) 
 590              foundKeys += 1 
 591          if 'pars' in kw: 
 592              self.set(pars=kw['pars']) 
 593              foundKeys += 1 
 594          if 'tdata' in kw: 
 595              tdata = kw['tdata'] 
 596              foundKeys += 1 
 597  #            print "tdata in kw of %s (%s):"%(self.name, type(self)), tdata
 
 598          else: 
 599              tdata = self.tdata 
 600  #            print "tdata from self of %s (%s):"%(self.name, type(self)), tdata
 
 601          if 'force' in kw: 
 602              force_overwrite = kw['force'] 
 603              foundKeys += 1 
 604          else: 
 605              force_overwrite = False 
 606          if len(kw) != foundKeys: 
 607              raise PyDSTool_KeyError('Invalid argument keys passed to compute()') 
 608          if tdata is None: 
 609              raise ValueError("tdata must be specified") 
 610          if len(tdata) == 1: 
 611              assert isinstance(tdata, float) or isinstance(tdata, int), \
 
 612                     'tdata must be either a single number or a pair' 
 613              t0_global = tdata[0] 
 614              t1_global = Inf 
 615          elif len(tdata) == 2: 
 616              t0_global = tdata[0] 
 617              t1_global = tdata[1] 
 618          else: 
 619              raise ValueError('tdata argument key may be either a single '
 
 620                                 'float or a pair of floats') 
 621          if not force_overwrite: 
 622              assert trajname not in self.trajectories, \
 
 623                                     'Trajectory name already exists' 
 624          assert self.modelInfo != {}, \
 
 625                 'No Generator or Model objects defined for this model' 
 626          return tdata, t0_global, t1_global, force_overwrite

 627  
 


628 -    def query(self, querykey=''):


629          """Return info about Model set-up.
 
 630          Valid query key: 'pars', 'parameters', 'events', 'submodels',
 
 631           'ics', 'initialconditions', 'vars', 'variables',
 
 632           'auxvars', 'auxvariables', 'vardomains', 'pardomains', 'abseps'
 
 633           """ 
 634          assert isinstance(querykey, str), \
 
 635                         ("Query argument must be a single string") 
 636          if querykey not in self._querykeys: 
 637              print 'Valid query keys are:', self._querykeys 
 638              print "('events' key only queries model-level events, not those" 
 639              print " inside sub-models)" 
 640              if querykey != '': 
 641                  raise TypeError('Query key '+querykey+' is not valid') 
 642          if querykey in ['pars', 'parameters']: 
 643              result = copy.copy(self.pars) 
 644          elif querykey in ['ics', 'initialconditions']: 
 645              result = copy.copy(self.icdict) 
 646          elif querykey == 'events': 
 647              result = {} 
 648              for dsName, model in self.registry.iteritems(): 
 649                  try: 
 650                      result.update(model.eventstruct.events) 
 651                  except AttributeError: 
 652                      # ds is a ModelInterface, not a Generator
 
 653                      result.update(model.query('events')) 
 654          elif querykey == 'submodels': 
 655              result = self.registry 
 656          elif querykey in ['vars', 'variables']: 
 657              result = copy.copy(self.allvars) 
 658          elif querykey in ['vardomains', 'xdomains']: 
 659              result = {} 
 660              # accumulate domains from each sub-model for regular variables
 
 661              for model in self.registry.values(): 
 662                  vardoms = model.query('vardomains') 
 663                  if len(result)==0: 
 664                      result.update(vardoms) 
 665                  else: 
 666                      for vname, vdom in result.iteritems(): 
 667                          if vdom.issingleton: 
 668                              # singleton
 
 669                              vdom_lo = vdom.get() 
 670                              vdom_hi = vdom_lo 
 671                          else: 
 672                              # range
 
 673                              vdom_lo = vdom[0] 
 674                              vdom_hi = vdom[1] 
 675                          if vardoms[vname][0] < vdom[0]: 
 676                              vdom[0] = vardoms[vname][0] 
 677                          if vardoms[vname][1] > vdom[1]: 
 678                              vdom[1] = vardoms[vname][1] 
 679                          if vdom._abseps < result[vname]._abseps: 
 680                              # have to keep abseps the tightest
 
 681                              # of any of the instances for safety
 
 682                              result[vname]._abseps = vdom._abseps 
 683                          result[vname] = vdom 
 684              # remaining vars are promoted aux vars
 
 685              for vname in remain(self.allvars, result.keys()): 
 686                  result[vname] = Interval(vname, float, [-Inf, Inf]) 
 687          elif querykey in ['pardomains', 'pdomains']: 
 688              result = {} 
 689              # accumulate domains from each sub-model for regular variables
 
 690              for model in self.registry.values(): 
 691                  pardoms = model.query('pardomains') 
 692                  if len(result)==0: 
 693                      result.update(pardoms) 
 694                  else: 
 695                      for pname, pdom in result.iteritems(): 
 696                          if pdom.issingleton: 
 697                              # singleton
 
 698                              pdom_lo = pdom.get() 
 699                              pdom_hi = pdom_lo 
 700                          else: 
 701                              # range
 
 702                              pdom_lo = pdom[0] 
 703                              pdom_hi = pdom[1] 
 704                          if pardoms[pname][0] < pdom[0]: 
 705                              pdom[0] = pardoms[pname][0] 
 706                          if pardoms[pname][1] > pdom[1]: 
 707                              pdom[1] = pardoms[pname][1] 
 708                          if pdom._abseps < result[pname]._abseps: 
 709                              # have to keep abseps the tightest
 
 710                              # of any of the instances for safety
 
 711                              result[pname]._abseps = pdom._abseps 
 712                          result[pname] = pdom 
 713          elif querykey in ['auxvars', 'auxvariables']: 
 714              result = copy.copy(self.auxvars) 
 715          elif querykey == 'abseps': 
 716              result = min([ds.query('abseps') for ds in self.registry.values()]) 
 717          return result

 718  
 


719 -    def getEventMappings(self, dsName):


720          try: 
 721              return self.modelInfo[dsName]['swRules'] 
 722          except KeyError: 
 723              raise NameError("Sub-model %s not found in model"%dsName)

 724  
 


725 -    def setPars(self, p, val):


726          # process multirefs first, then hierarchical names
 
 727          # calls itself recursively to resolve all names
 
 728          if isMultiRef(p): 
 729              # e.g to change a group of numerically indexed parameter names
 
 730              # such as p0 - p9 or comp1.p0.g - comp1.p9.g
 
 731              # extract numeric range of pars
 
 732              # [ and ] are guaranteed to be present, from isMultiRef()
 
 733              lbrace = p.find('[') 
 734              rbrace = p.find(']') 
 735              if rbrace < lbrace: 
 736                  raise ValueError("Invalid multiple reference to pars") 
 737              rootname = p[:lbrace] 
 738              rangespec = p[lbrace+1:rbrace].split(',') 
 739              try: 
 740                  remainder = p[rbrace+1:] 
 741              except KeyError: 
 742                  # no more of p after this multireference
 
 743                  remainder = '' 
 744              if len(rangespec) != 2: 
 745                  raise ValueError("Invalid multiple reference to pars") 
 746              loix = int(rangespec[0]) 
 747              hiix = int(rangespec[1]) 
 748              if loix >= hiix: 
 749                  raise ValueError("Invalid multiple reference to pars") 
 750              # call setPars for each resolved name (these may include further
 
 751              # multi references or hierarchical names
 
 752              for ix in range(loix, hiix+1): 
 753                  self.setPars(rootname+str(ix), val) 
 754          elif isHierarchicalName(p): 
 755              if self._mspecdict is None: 
 756                  raise PyDSTool_ExistError("Cannot use this functionality for"
 
 757                                      " models not defined through ModelSpec") 
 758              # find out: is root of p a valid 'type' in model spec?
 
 759              # find all occurrences of last p
 
 760              allFoundNames = [] 
 761              for mspecinfo in self._mspecdict.values(): 
 762                  foundNames = searchModelSpec(mspecinfo['modelspec'], p) 
 763                  # don't add duplicates
 
 764                  allFoundNames.extend(remain(foundNames,allFoundNames)) 
 765              # if allFoundNames == [] then either the hierarchical name was
 
 766              # a specific reference, or the type matching is invalid.
 
 767              # either way, we can just call set(p) and let that resolve the issue
 
 768              # (note that all multi-refs will have been dealt with by this point)
 
 769              if allFoundNames == []: 
 770                  self.set(pars={p: val}) 
 771              else: 
 772                  self.set(pars={}.fromkeys(allFoundNames, val)) 
 773          else: 
 774              self.set(pars={p: val})

 775  
 
 776  
 


777 -    def setICs(self, p, val):


778          # process multirefs first, then hierarchical names
 
 779          # calls itself recursively to resolve all names
 
 780          if isMultiRef(p): 
 781              # e.g to change a group of numerically indexed parameter names
 
 782              # such as p0 - p9 or comp1.p0.g - comp1.p9.g
 
 783              # extract numeric range of pars
 
 784              # [ and ] are guaranteed to be present, from isMultiRef()
 
 785              lbrace = p.find('[') 
 786              rbrace = p.find(']') 
 787              if rbrace < lbrace: 
 788                  raise ValueError("Invalid multiple reference to initial conditions") 
 789              rootname = p[:lbrace] 
 790              rangespec = p[lbrace+1:rbrace].split(',') 
 791              try: 
 792                  remainder = p[rbrace+1:] 
 793              except KeyError: 
 794                  # no more of p after this multireference
 
 795                  remainder = '' 
 796              if len(rangespec) != 2: 
 797                  raise ValueError("Invalid multiple reference to initial conditions") 
 798              loix = int(rangespec[0]) 
 799              hiix = int(rangespec[1]) 
 800              if loix >= hiix: 
 801                  raise ValueError("Invalid multiple reference to initial conditions") 
 802              # call setICs for each resolved name (these may include further
 
 803              # multi references or hierarchical names
 
 804              for ix in range(loix, hiix+1): 
 805                  self.setICs(rootname+str(ix), val) 
 806          elif isHierarchicalName(p): 
 807              if self._mspecdict is None: 
 808                  raise PyDSTool_ExistError("Cannot use this functionality for"
 
 809                                      " models not defined through ModelSpec") 
 810              # find out: is root of p a valid 'type' in model spec?
 
 811              # find all occurrences of last p
 
 812              allFoundNames = [] 
 813              for mspecinfo in self._mspecdict.values(): 
 814                  foundNames = searchModelSpec(mspecinfo['modelspec'], p) 
 815                  # don't add duplicates
 
 816                  allFoundNames.extend(remain(foundNames,allFoundNames)) 
 817              # if allFoundNames == [] then either the hierarchical name was
 
 818              # a specific reference, or the type matching is invalid.
 
 819              # either way, we can just call set(p) and let that resolve the issue
 
 820              # (note that all multi-refs will have been dealt with by this point)
 
 821              if allFoundNames == []: 
 822                  self.set(ics={p: val}) 
 823              else: 
 824                  self.set(ics={}.fromkeys(allFoundNames, val)) 
 825          else: 
 826              self.set(ics={p: val})

 827  
 
 828  
 


829 -    def set(self, **kw):


830          """Set specific parameters of Model. These will get passed on to
 
 831          all Generators/sub-models that support these keys unless the
 
 832          restrictDSList argument is set (only applies to keys algparams,
 
 833          checklevel, and abseps).
 
 834  
 
 835          Permitted keys: 'pars', 'algparams', 'checklevel', 'abseps',
 
 836                          'ics', 'inputs', 'tdata', 'restrictDSlist',
 
 837                          'globalt0', 'verboselevel', 'inputs_t0'
 
 838          """ 
 839          for key in kw: 
 840              if key not in self._setkeys: 
 841                  raise KeyError("Not a permitted parameter argument: %s"%key + \
 
 842                                      ". Allowed keys: "+str(self._setkeys)) 
 843          if 'restrictDSlist' in kw: 
 844              restrictDSlist = kw['restrictDSlist'] 
 845          else: 
 846              restrictDSlist = [] 
 847          # Handle initial conditions here, because compute will pass
 
 848          # the values on to the appropriate sub-models when they are called.
 
 849          if 'ics' in kw: 
 850              self.icdict.update(filteredDict(dict(kw['ics']), self.icdict.keys())) 
 851          if 'tdata' in kw: 
 852              self.tdata = kw['tdata'] 
 853          if 'abseps' in kw: 
 854              self._abseps = kw['abseps'] 
 855          if 'inputs_t0' in kw: 
 856              for model in self.registry.values(): 
 857                  # Propagate values to sub-models.
 
 858                  # If any values are strings they must refer to a parameter, so
 
 859                  # here we evaluate them. We keep a record of these for later
 
 860                  # automated updating.
 
 861                  t0val_dict = kw['inputs_t0'] 
 862                  for inp, val in t0val_dict.items(): 
 863                      if isinstance(val, str): 
 864                          try: 
 865                              new_val = self.pars[val] 
 866                          except KeyError: 
 867                              raise ValueError("Input t0 to parameter link "
 
 868                                               "invalid: no such parameter "+val) 
 869                          t0val_dict[inp] = new_val 
 870                          self._inputt0_par_links.update({inp: val}) 
 871                      elif isinstance(val, _num_types): 
 872                          if inp in self._inputt0_par_links: 
 873                              # no longer parameter-linked
 
 874                              del self._inputt0_par_links[inp] 
 875                      else: 
 876                          raise TypeError("Invalid type for input t0 value") 
 877                  try: 
 878                      model.set(inputs_t0=t0val_dict) 
 879                  except AssertionError: 
 880                      # generator doesn't involve inputs
 
 881                      pass 
 882          if 'verboselevel' in kw: 
 883              if kw['verboselevel'] in [0,1,2]: 
 884                  self.verboselevel = kw['verboselevel'] 
 885              else: 
 886                  raise ValueError("Verbosity level value must be 0, 1, or 2") 
 887              for model in self.registry.values(): 
 888                  # propagate to sub-models
 
 889                  try: 
 890                      model.set(verboselevel=self.verboselevel) 
 891                  except KeyError: 
 892                      # generator doesn't support verboselevel
 
 893                      pass 
 894          if restrictDSlist == []: 
 895              restrictDSlist = self.registry.keys() 
 896          # For the remaining keys, must propagate parameter changes to all
 
 897          # sub-models throughout modelInfo structure.
 
 898          #
 
 899          # Changed by WES 10FEB06 to handle problem of 'new' pars being
 
 900          # added if the parameter names do not exist in any generators
 
 901          dsis = self.modelInfo.values() 
 902          numDSs = len(dsis) 
 903          # loop over keywords
 
 904          for key, value in filteredDict(kw, ['ics', 'tdata',
 
 905                              'inputs_t0', 'restrictDSlist', 'globalt0'],
 
 906                                  neg=True).iteritems(): 
 907              # keep track of the number of errors on this keyword
 
 908              if isinstance(value, dict): 
 909                  # keep track of entry errors for this key
 
 910                  entry_err_attr = {} 
 911                  entry_err_val = {} 
 912                  for entrykey, entryval in value.iteritems(): 
 913                      entry_err_attr[entrykey] = 0 
 914                      entry_err_val[entrykey] = 0 
 915              else: 
 916                  entry_err_attr = 0 
 917                  entry_err_val = 0 
 918              # try setting pars in each sub-model
 
 919              for infodict in dsis: 
 920                  callparsDict = {} 
 921                  # select out the ones relevant to this sub-model
 
 922                  ds = infodict['dsi'].model 
 923                  if hasattr(ds, '_validKeys'): 
 924                      options = ds._validKeys 
 925                  elif hasattr(ds, '_setkeys'): 
 926                      options = ds._setkeys 
 927                  if key in options: 
 928                      if key in ['algparams', 'checklevel', 'abseps'] and \
 
 929                          ds.name not in restrictDSlist: 
 930                              # only apply these keys to the restricted list
 
 931                              continue 
 932                      if isinstance(value, dict): 
 933                          for entrykey, entryval in value.iteritems(): 
 934                              try: 
 935                                  ds.set(**{key:{entrykey:entryval}}) 
 936                              except PyDSTool_AttributeError: 
 937                                  entry_err_attr[entrykey] += 1 
 938                              except PyDSTool_ValueError: 
 939                                  entry_err_val[entrykey] += 1 
 940                              except AssertionError: 
 941                                  # key not valid for this type of ds
 
 942                                  pass 
 943                      else: 
 944                          try: 
 945                              ds.set(**{key: value}) 
 946                          except PyDSTool_AttributeError: 
 947                              entry_err_attr += 1 
 948                          except PyDSTool_ValueError: 
 949                              entry_err_val += 1 
 950                          except AssertionError: 
 951                              # key not valid for this type of ds
 
 952                              pass 
 953              # Check that none of the entries in the dictionary caused errors
 
 954              # in each sub-model
 
 955              if isinstance(value, dict): 
 956                  for entrykey, entryval in value.iteritems(): 
 957                      if entry_err_attr[entrykey] == numDSs: 
 958                          raise PyDSTool_AttributeError('Parameter does not' +\
 
 959                                ' exist in any sub-model: %s = %f'%(entrykey,
 
 960                                                                    entryval)) 
 961                      if entry_err_val[entrykey] == numDSs: 
 962                          raise PyDSTool_ValueError('Parameter value error in' +\
 
 963                                ' every sub-model: %s = %f'%(entrykey, entryval)) 
 964                      else: 
 965                          # can't think of other ways for this error to crop up
 
 966                          pass 
 967              else: 
 968                  if entry_err_attr == numDSs: 
 969                      raise PyDSTool_AttributeError('Parameter does not exist' +\
 
 970                               ' in any sub-model: %s'%key) 
 971                  if entry_err_val == numDSs: 
 972                      raise PyDSTool_ValueError('Parameter value error in' +\
 
 973                                ' every sub-model: %s'%key) 
 974              del(entry_err_attr) 
 975              del(entry_err_val) 
 976          self._generateParamInfo()

 977  
 


978 -    def __getitem__(self, trajname):


979          try: 
 980              return self.trajectories[trajname] 
 981          except KeyError: 
 982              raise ValueError('No such trajectory.')

 983  
 


984 -    def __delitem__(self, trajname):


985          self._delTraj(trajname)

 986  
 


987 -    def _delTraj(self, trajname):


988          """Delete a named trajectory from the database.""" 
 989          try: 
 990              traj = self.trajectories[trajname] 
 991          except KeyError: 
 992              # a trajectory piece may have been created without
 
 993              # the top-level trajectory ever being completed
 
 994              # (e.g. after an unxpected error or ^C interruption)
 
 995              ##raise ValueError('No such trajectory.')
 
 996              l = len(trajname) 
 997              for m in self.registry.values(): 
 998                  # delete all matching pieces (of form trajname + '_' + <digits>)
 
 999                  for n in m.trajectories.keys(): 
1000                      if n[:l] == trajname and n[l] == '_' and n[l+1:].isdigit(): 
1001                          m._delTraj(trajname) 
1002          else: 
1003              # propagate deletions down through registry
 
1004              if not isinstance(traj.modelNames, str): 
1005                  for i, model_name_i in enumerate(traj.modelNames): 
1006                      del(self.registry[model_name_i][trajname+'_%i'%i]) 
1007              del(self.trajectories[trajname])

1008  
 
1009  
 


1010 -    def __call__(self, trajname, t, coords=None, asGlobalTime=True,
 
1011                   asmap=False):


1012          """Evaluate position of hybrid trajectory at time t.
 
1013          if optional argument asmap == True then t must be an integer in
 
1014          [0, #segments].
 
1015          """ 
1016          try: 
1017              traj = self.trajectories[trajname] 
1018          except KeyError: 
1019              raise ValueError("trajectory '"+trajname+"' unknown") 
1020          else: 
1021              return traj(t, coords=coords, asGlobalTime=asGlobalTime,
 
1022                          asmap=asmap)

1023  
 
1024  
 


1025 -    def getEndPoint(self, trajname, end=1):


1026          """Returns endpoint of specified trajectory as Point.
 
1027          trajname: name of selected trajectory
 
1028          end: (default=1) index of trajectory endpoint.
 
1029          0 => first, 1 => last
 
1030          """ 
1031          xdict = {} 
1032          if end not in [0,1]: 
1033              raise ValueError("end must be 0, 1") 
1034          endtraj = self.trajectories[trajname].trajSeq[-end] 
1035          for xname in endtraj.coordnames: 
1036              try: 
1037                  xdict[endtraj._FScompatibleNamesInv(xname)] = \
 
1038                       endtraj.variables[xname].output.datapoints[1][-end] 
1039              except KeyError: 
1040                  # auxiliary var didn't need calling
 
1041                  pass 
1042              except AttributeError: 
1043                  # non-point based output attributes of a Variable need
 
1044                  # to be called ...
 
1045                  tend = endtraj.indepdomain[end] 
1046                  xdict[endtraj._FScompatibleNamesInv(xname)] = \
 
1047                       endtraj.variables[xname](tend) 
1048              except PyDSTool_BoundsError: 
1049                  print "Value out of bounds in variable call:" 
1050                  print "  variable '%s' was called at time %f"%(xname, tend) 
1051                  raise 
1052          return Point({'coorddict': xdict,
 
1053                'coordnames': endtraj._FScompatibleNamesInv(endtraj.coordnames),
 
1054                'coordtype': float,
 
1055                'norm': self._normord})

1056  
 


1057 -    def getEndTime(self, trajname, end=1):


1058          """Returns end time of specified trajectory.
 
1059          trajname: name of selected trajectory
 
1060          end: (default=1) index of trajectory endpoint.
 
1061          0 => first, 1 => last
 
1062          """ 
1063          if end not in [0,1]: 
1064              raise ValueError("end must be 0, 1") 
1065          endtraj = self.trajectories[trajname].trajSeq[-end] 
1066          tend = endtraj.indepdomain[end] 
1067          return tend

1068  
 


1069 -    def _validateVarNames(self, names):


1070          """Check types and uniqueness of variable names.""" 
1071          namelist = []  # records those seen so far 
1072          for vname in names: 
1073              assert isinstance(vname, str), 'variable name must be a string' 
1074              assert vname not in namelist, ('variable names must be unique for'
 
1075                                             ' model') 
1076              namelist.append(vname)

1077  
 
1078  
 


1079 -    def forceObsVars(self, varnames):


1080          """Force variables to be the observables in the Model.
 
1081          May also promote auxiliary variables.""" 
1082          r = remain(varnames, self.obsvars+self.intvars+self.auxvars) 
1083          if len(r) > 0: 
1084              # then there are names given that are not known as
 
1085              # obs, int, or aux
 
1086              raise ValueError("Unknown variable names: "+str(r)) 
1087          for v in remain(varnames, self.obsvars): 
1088              # only include names that are not already observables
 
1089              self.obsvars.append(v) 
1090          # remove any vars that are now observables
 
1091          self.intvars = remain(self.intvars, varnames) 
1092          self.auxvars = remain(self.auxvars, varnames) 
1093          self.obsvars.sort() 
1094          self.intvars.sort() 
1095          self.auxvars.sort() 
1096          self.allvars = self.obsvars + self.intvars 
1097          self.allvars.sort() 
1098          self.dimension = len(self.allvars)

1099  
 
1100  
 


1101 -    def forceIntVars(self, varnames):


1102          """Force variables to become internal variables in the Model.
 
1103          May also promote auxiliary variables.""" 
1104          r = remain(varnames, self.obsvars+self.intvars+self.auxvars) 
1105          if len(r) > 0: 
1106              # then there are names given that are not known as
 
1107              # obs, int, or aux
 
1108              raise ValueError("Unknown variable names: "+str(r)) 
1109          for v in remain(varnames, self.intvars): 
1110              # only include names that are not already internals
 
1111              self.intvars.append(v) 
1112          # remove any vars that are now internals
 
1113          self.obsvars = remain(self.obsvars, varnames) 
1114          self.auxvars = remain(self.auxvars, varnames) 
1115          self.obsvars.sort() 
1116          self.intvars.sort() 
1117          self.auxvars.sort() 
1118          self.allvars = self.obsvars + self.intvars 
1119          self.allvars.sort() 
1120          self.dimension = len(self.allvars)

1121  
 
1122  
 


1123 -    def defaultVars(self):


1124          """(Re)set to default observable and internal variable names.""" 
1125          obsvars, intvars, auxvars = self._makeDefaultVarNames() 
1126          self._validateVarNames(obsvars + intvars + auxvars) 
1127          # OK to store these permanently after varname validation
 
1128          self.obsvars = obsvars 
1129          self.intvars = intvars 
1130          self.auxvars = auxvars 
1131          self.obsvars.sort() 
1132          self.intvars.sort() 
1133          self.auxvars.sort() 
1134          self.allvars = self.obsvars + self.intvars 
1135          self.allvars.sort() 
1136          self.dimension = len(self.allvars)

1137  
 
1138  
 


1139 -    def info(self, verboselevel=1):


1140          print self._infostr(verboselevel)

1141  
 
1142  
 


1143 -    def __repr__(self):


1144          return self._infostr(verbose=0)

1145  
 
1146      __str__ = __repr__ 
1147  
 
1148  
 


1149 -    def __copy__(self):


1150          pickledself = pickle.dumps(self) 
1151          return pickle.loads(pickledself)

1152  
 
1153  
 


1154 -    def __deepcopy__(self, memo=None, _nil=[]):


1155          pickledself = pickle.dumps(self) 
1156          return pickle.loads(pickledself)

1157  
 
1158  
 


1159 -    def renameTraj(self, trajname, newname, force=False):


1160          """Rename stored trajectory. Force option (default False)
 
1161          will overwrite any existing trajectory with the new name.
 
1162          """ 
1163          try: 
1164              traj = self.trajectories[trajname] 
1165          except KeyError: 
1166              raise ValueError('No such trajectory name %s'%trajname) 
1167          if trajname != newname: 
1168              if newname not in self.trajectories or force: 
1169                  self.trajectories[newname] = traj 
1170                  del self.trajectories[trajname] 
1171                  traj.name = newname 
1172              else: 
1173                  raise ValueError("Name %s already exists"%newname)

1174  
 


1175 -    def getTrajModelName(self, trajname, t=None):


1176          """Return the named trajectory's associated sub-model(s) used
 
1177          to create it, specific to time t if given.""" 
1178          try: 
1179              modelNames = self.trajectories[trajname].modelNames 
1180          except KeyError: 
1181              raise ValueError('No such trajectory name %s'%trajname) 
1182          if t is None: 
1183              # return list of Generators for associated hybrid trajectory
 
1184              return modelNames 
1185          else: 
1186              parts = self.getTrajTimePartitions(trajname) 
1187              pix = 0 
1188              for pinterval in parts: 
1189                  if pinterval.contains(t) is not notcontained: 
1190                      return modelNames[pix] 
1191                  else: 
1192                      pix += 1

1193  
 


1194 -    def getTrajEventTimes(self, trajname, events=None):


1195          """Return the named trajectory's Generator-flagged event times.
 
1196  
 
1197          events argument can be singleton string name of an event,
 
1198          returning the event data, or events can be a list of event names,
 
1199          returning a dictionary of event name -> event data.
 
1200          Event names should use hierarchical naming convention, if
 
1201          applicable.""" 
1202          try: 
1203              return self.trajectories[trajname].getEventTimes(events) 
1204          except KeyError: 
1205              raise

1206              #raise ValueError('No such trajectory name')
 
1207  
 


1208 -    def getTrajEvents(self, trajname, events=None):


1209          """Return the named trajectory's Generator-flagged events.
 
1210  
 
1211          events argument can be singleton string name of an event,
 
1212          returning the event data, or events can be a list of event names,
 
1213          returning a dictionary of event name -> event data.
 
1214          Event names should use hierarchical naming convention, if
 
1215          applicable.""" 
1216          try: 
1217              return self.trajectories[trajname].getEvents(events) 
1218          except KeyError: 
1219              raise ValueError('No such trajectory name')

1220  
 


1221 -    def getTrajEventStruct(self, trajname):


1222          """Return the named trajectory's model event structure (representing
 
1223          external constraints), as present when it was computed.
 
1224          """ 
1225          try: 
1226              return self.trajectories[trajname].modelEventStructs 
1227          except KeyError: 
1228              raise ValueError('No such trajectory name')

1229  
 


1230 -    def getTrajTimeInterval(self, trajname):


1231          """Return the named trajectory's time domain,
 
1232          over which it is defined, in a single interval.""" 
1233          try: 
1234              return self.trajectories[trajname].indepdomain 
1235          except KeyError: 
1236              raise ValueError('No such trajectory name')

1237  
 


1238 -    def getTrajTimePartitions(self, trajname):


1239          """Return the named trajectory's time domain,
 
1240          over which it is defined, as a list of time interval partitions.""" 
1241          try: 
1242              return self.trajectories[trajname].timePartitions 
1243          except KeyError: 
1244              raise ValueError('No such trajectory name')

1245  
 


1246 -    def getDSAlgPars(self, target, par, idx=None):


1247          """
 
1248          Returns value of given algorithmic parameter for selected sub-model.
 
1249          target -- name of sub-model in model (cannot be list).
 
1250          par -- name of algorithmic parameter.
 
1251          idx -- (optional) index into value if algorithmic parameter val is a
 
1252            list of values.
 
1253          """ 
1254          if target in self.registry.keys(): 
1255              algpars = self.registry[target].get('algparams') 
1256              if par in algpars.keys(): 
1257                  if isinstance(algpars[par], list): 
1258                      if idx is not None: 
1259                          if isinstance(idx, list): 
1260                              val = [algpars[par][x] for x in idx] 
1261                          else: 
1262                              val = algpars[par][idx] 
1263                      else: 
1264                          val = algpars[par] 
1265                  else: 
1266                      val = algpars[par] 
1267              else: 
1268                  val = None 
1269          else: 
1270              raise ValueError("Target sub-model name not found") 
1271          return val

1272  
 


1273 -    def setDSAlgPars(self, target, par, val):


1274          """
 
1275          Set value of algorithmic parameter in a specific generator.
 
1276          target -- name or list of generators in model.
 
1277          par -- name of algorithmic parameter is to be set.
 
1278          val -- value to which the algorithmic parameter is to be set.
 
1279  
 
1280          if target is a list, then algorithmic pararameter 'par' is
 
1281          set to 'val' for every generator in the list, if par exists for that
 
1282          generator.
 
1283  
 
1284          WARNING: THIS FUNCTION IS NOT 'SAFE' -- IT DOES NOT CHECK THAT VALS
 
1285            ARE APPROPRIATE TO PARAMETERS!!!
 
1286          """ 
1287  
 
1288          if isinstance(target, list): 
1289              subModelList = target 
1290          else: 
1291              subModelList = [target] 
1292          for dsName in subModelList: 
1293              algpars = self.registry[dsName].get('algparams') 
1294              if par in algpars.keys(): 
1295                  # If target is a list, make sure that the input list is of the
 
1296                  # appropriate length, otherwise warn and skip
 
1297                  if isinstance(algpars[par], list): 
1298                      if isinstance(val, list): 
1299                          if len(algpars[par]) != len(val): 
1300                              print "Warning: par %s list len (%d) in generator %s doesn't match val list len (%d). Skipping."%(par, len(algpars[par]), dsName, len(val)) 
1301                              continue 
1302                          else: 
1303                              algpars[par] = val 
1304                      else: 
1305                          # Set every member of the list to that value
 
1306                          for x in range(len(algpars[par])): 
1307                              algpars[par][x] = val 
1308                  else: 
1309                      if isinstance(val, list): 
1310                          print "Warning: par %s type (%s) in generator %s doesn't match val type (%s). Skipping."%(par, type(algpars[par]), dsName, type(val)) 
1311                      else: 
1312                          algpars[par] = val 
1313              else: 
1314                  pass

1315  
 
1316  
 


1317 -    def sample(self, trajname, coords=None, dt=None,
 
1318                     tlo=None, thi=None, doEvents=True, precise=False):


1319          """Uniformly sample the named trajectory over range indicated,
 
1320          including any event points. (e.g. use this call for plotting purposes.)
 
1321          Outputs a Pointset from the trajectory over a given range.
 
1322  
 
1323          Arguments:
 
1324  
 
1325            trajname   Name of stored trajectory to sample
 
1326            coords     (optional) list of variable names to include in output
 
1327            dt         (optional) step size to use in sampling of independent variable
 
1328                       If not given, the underlying time mesh is used, if available.
 
1329            tlo        (optional) Start value for independent variable, default 0
 
1330            thi        (optional) End value for independent variable, default last value
 
1331            doEvents   (optional) include any event points in output, default True
 
1332            precise    (optional) The default value, False, causes an attempt to use
 
1333                       the underlying mesh of the trajectory to return a Pointset more
 
1334                       quickly. Currently, this can only be used for trajectories that
 
1335                       have a single segment (non-hybrid).
 
1336          """ 
1337          try: 
1338              return self.trajectories[trajname].sample(coords, dt, tlo, thi,
 
1339                                                    doEvents, precise) 
1340          except KeyError: 
1341              raise

1342  
 
1343  
 


1344 -    def _infostr(self, dsName=None, verbosity=0):


1345          """Return string information about named sub-model (if given by dsName)
 
1346          at given verbosity level (default 0)""" 
1347          pp = pprint.PrettyPrinter(indent=3) 
1348          if dsName is None: 
1349              result = {} 
1350              res_str = "Sub-models defined in model %s:\n" % self.name 
1351              for name, infodict in self.modelInfo.iteritems(): 
1352                  result[name] = infodict['dsi'].model._infostr(verbosity) 
1353              return res_str + pp.pprint(result) 
1354          else: 
1355              # return more information for a single sub-model
 
1356              try: 
1357                  result = {dsName: self.modelInfo[dsName]['dsi'].model._infostr(verbosity)+"\n"+\
 
1358                        "Event mapping info:\n" + str(self.modelInfo[dsName]['swRules'])} 
1359              except KeyError: 
1360                  raise NameError("Sub-model %s not found in model"%dsName) 
1361              return "Sub-model %s:\n" % (dsName) + pp.pprint(result)

1362  
 


1363 -    def showDSEventInfo(self, target, verbosity=1, ics=None, t=0):


1364          # call to eventstruct prints info to stdout
 
1365          if ics is None: 
1366              ics = self.icdict 
1367          estruct = self.modelInfo[target]['dsi'].get('eventstruct', ics, t) 
1368          estruct(verbosity)

1369  
 


1370 -    def getDSEventTerm(self, target, flagVal=True, ics=None, t=0):


1371          """
 
1372          List of events in target which are terminal/non-terminal according to
 
1373          value of flagVal.
 
1374          target -- name of generators in model (cannot be list)
 
1375          flagVal -- True (terminal) or False (non-terminal)
 
1376          """ 
1377          if ics is None: 
1378              ics = self.icdict 
1379          estruct = self.modelInfo[target]['dsi'].get('eventstruct', ics, t) 
1380          if isinstance(target, str): 
1381              if flagVal: 
1382                  return [x[0] for x in estruct.getTermEvents()] 
1383              else: 
1384                  return [x[0] for x in estruct.getNonTermEvents()] 
1385          else: 
1386              return []

1387  
 


1388 -    def setDSEventTerm(self, target, eventTarget, flagVal, ics=None, t=0):


1389          """
 
1390          Set event in a specific generator to be (non)terminal.
 
1391          target -- name or list of names of generators in model.
 
1392          eventTarget -- name or list of names of events in specified generator(s)
 
1393          flagVal -- True (event terminal) or False (event non-terminal)
 
1394  
 
1395          flagVal is applied to every listed event in every listed generator, if
 
1396            events and generators exist.
 
1397          """ 
1398          if ics is None: 
1399              ics = self.icdict 
1400          if isinstance(target, list): 
1401              for targName in target: 
1402                  estruct = self.modelInfo[targName]['dsi'].get('eventstruct', ics, t) 
1403                  estruct.setTermFlag(eventTarget, flagVal) 
1404          else: 
1405              estruct = self.modelInfo[target]['dsi'].get('eventstruct', ics, t) 
1406              estruct.setTermFlag(eventTarget, flagVal)

1407  
 


1408 -    def getDSEventActive(self, target, flagVal=True, ics=None, t=0):


1409          """
 
1410          List of events in target which are active/inactive according to
 
1411          value of flagVal.
 
1412          target -- name of generators in model (cannot be list)
 
1413          flagVal -- True (active) or False (inactive)
 
1414          """ 
1415          if ics is None: 
1416              ics = self.icdict 
1417          estruct = self.modelInfo[target]['dsi'].get('eventstruct', ics, t) 
1418          if isinstance(target, str): 
1419              if flagVal: 
1420                  return [x[0] for x in estruct.getActiveEvents()] 
1421              else: 
1422                  return [x[0] for x in estruct.getNonActiveEvents()] 
1423          else: 
1424              return []

1425  
 


1426 -    def setDSEventActive(self, target, eventTarget, flagVal, ics=None, t=0):


1427          """
 
1428          Set event in a specific generator to be (in)active.
 
1429          target -- name or list of names of generators in model.
 
1430          eventTarget -- name or list of names of events in specified generator(s)
 
1431          flagVal -- True (event active) or False (event inactive)
 
1432  
 
1433          flagVal is applied to every listed event in every listed generator, if
 
1434          events and generators exist.
 
1435          """ 
1436          if ics is None: 
1437              ics = self.icdict 
1438          if isinstance(target, list): 
1439              for targName in target: 
1440                  estruct = self.modelInfo[targName]['dsi'].get('eventstruct', ics, t) 
1441                  estruct.setActiveFlag(eventTarget, flagVal) 
1442          else: 
1443              estruct = self.modelInfo[target]['dsi'].get('eventstruct', ics, t) 
1444              estruct.setActiveFlag(eventTarget, flagVal)

1445  
 


1446 -    def setDSEventICs(self, target, eventTarget, val, ics=None, t=0):


1447          """
 
1448          Set event in a specific generator to have specified initial conditions.
 
1449          target -- name or list of names of generators in model.
 
1450          eventTarget -- name or list of names of events in specified generator(s)
 
1451          val -- dictionary of varnames and initial condition values
 
1452  
 
1453          val is applied to every listed event in every listed generator, if
 
1454          events and generators exist.
 
1455          If a varname in the val dict does not exist in a specified
 
1456          generator/event, then it is skipped.
 
1457          """ 
1458          if ics is None: 
1459              ics = self.icdict 
1460          if isinstance(target, list): 
1461              for targName in target: 
1462                  estruct = self.modelInfo[targName]['dsi'].get('eventstruct', ics, t) 
1463                  estruct.setICs(eventTarget, val) 
1464          else: 
1465              estruct = self.modelInfo[target]['dsi'].get('eventstruct', ics, t) 
1466              estruct.setICs(eventTarget, val)

1467  
 


1468 -    def setDSEventPrecise(self, target, eventTarget, flagVal, ics=None, t=0):


1469          """
 
1470          Set event in a specific generator to be (im)precise.
 
1471          target -- name or list of names of generators in model.
 
1472          eventTarget -- name or list of names of events in specified generator(s)
 
1473          flagVal -- True (event precise) or False (event imprecise)
 
1474  
 
1475          flagVal is applied to every listed event in every listed generator, if events and generators exist.
 
1476          """ 
1477          if ics is None: 
1478              ics = self.icdict 
1479          if isinstance(target, list): 
1480              for targName in target: 
1481                  estruct = self.modelInfo[targName]['dsi'].get('eventstruct', ics, t) 
1482                  estruct.setPreciseFlag(eventTarget, flagVal) 
1483          else: 
1484              estruct = self.modelInfo[target]['dsi'].get('eventstruct', ics, t) 
1485              estruct.setPreciseFlag(eventTarget, flagVal)

1486  
 


1487 -    def setDSEventDelay(self, target, eventTarget, val, ics=None, t=0):


1488          """
 
1489          Set event in a specific generator to have specified event delay.
 
1490          target -- name or list of names of generators in model.
 
1491          eventTarget -- name or list of names of events in specified generator(s)
 
1492          val -- event delay (int, float >= 0)
 
1493  
 
1494          val is applied to every listed event in every listed generator, if events and generators exist.
 
1495          """ 
1496          if ics is None: 
1497              ics = self.icdict 
1498          if isinstance(target, list): 
1499              for targName in target: 
1500                  estruct = self.modelInfo[targName]['dsi'].get('eventstruct', ics, t) 
1501                  estruct.setEventDelay(eventTarget, val) 
1502          else: 
1503              estruct = self.modelInfo[target]['dsi'].get('eventstruct', ics, t) 
1504              estruct.setEventDelay(eventTarget, val)

1505  
 


1506 -    def setDSEventInterval(self, target, eventTarget, val, ics=None, t=0):


1507          """
 
1508          Set event in a specific generator to have specified event interval.
 
1509          target -- name or list of names of generators in model.
 
1510          eventTarget -- name or list of names of events in specified generator(s)
 
1511          val -- event interval (int, float >= 0)
 
1512  
 
1513          val is applied to every listed event in every listed generator, if events and generators exist.
 
1514          """ 
1515          if ics is None: 
1516              ics = self.icdict 
1517          if isinstance(target, list): 
1518              for targName in target: 
1519                  estruct = self.modelInfo[targName]['dsi'].get('eventstruct', ics, t) 
1520                  estruct.setEventInterval(eventTarget, val) 
1521          else: 
1522              estruct = self.modelInfo[target]['dsi'].get('eventstruct', ics, t) 
1523              estruct.setEventInterval(eventTarget, val)

1524  
 


1525 -    def setDSEventTol(self, target, eventTarget, val, ics=None, t=0):


1526          """
 
1527          Set event in a specific generator to have specified event tolerance.
 
1528          target -- name or list of names of generators in model.
 
1529          eventTarget -- name or list of names of events in specified generator(s)
 
1530          val -- event tolerance (int, float >= 0)
 
1531  
 
1532          val is applied to every listed event in every listed generator, if events and generators exist.
 
1533          """ 
1534          if ics is None: 
1535              ics = self.icdict 
1536          if isinstance(target, list): 
1537              for targName in target: 
1538                  estruct = self.modelInfo[targName]['dsi'].get('eventstruct', ics, t) 
1539                  estruct.setEventTol(eventTarget, val) 
1540          else: 
1541              estruct = self.modelInfo[target]['dsi'].get('eventstruct', ics, t) 
1542              estruct.setEventTol(eventTarget, val)

1543  
 


1544 -    def setDSEventDir(self, target, eventTarget, val, ics=None, t=0):


1545          """
 
1546          Set event in a specific generator to have specified direction code.
 
1547          target -- name or list of names of generators in model.
 
1548          eventTarget -- name or list of names of events in specified generator(s)
 
1549          val -- direction code (-1: decreasing, 1: increasing, 0: either direction)
 
1550  
 
1551          val is applied to every listed event in every listed generator, if events and generators exist.
 
1552          """ 
1553          if ics is None: 
1554              ics = self.icdict 
1555          if isinstance(target, list): 
1556              for targName in target: 
1557                  estruct = self.modelInfo[targName]['dsi'].get('eventstruct', ics, t) 
1558                  estruct.setEventDir(eventTarget, val) 
1559          else: 
1560              estruct = self.modelInfo[target]['dsi'].get('eventstruct', ics, t) 
1561              estruct.setEventDir(eventTarget, val)

1562  
 


1563 -    def setDSEventStartTime(self, target, eventTarget, val, ics=None, t=0):


1564          """
 
1565          Set event in a specific generator to have specified start time.
 
1566          target -- name or list of names of generators in model.
 
1567          eventTarget -- name or list of names of events in specified generator(s)
 
1568          val -- start time (float, int)
 
1569  
 
1570          val is applied to every listed event in every listed generator, if events and generators exist.
 
1571          """ 
1572          if ics is None: 
1573              ics = self.icdict 
1574          if isinstance(target, list): 
1575              for targName in target: 
1576                  estruct = self.modelInfo[targName]['dsi'].get('eventstruct', ics, t) 
1577                  estruct.setStartTime(eventTarget, val) 
1578          else: 
1579              estruct = self.modelInfo[target]['dsi'].get('eventstruct', ics, t) 
1580              estruct.setStartTime(eventTarget, val)

1581  
 


1582 -    def setDSEventBisect(self, target, eventTarget, val, ics=None, t=0):


1583          """
 
1584          Set event in a specific generator to have specified bisect limit.
 
1585          target -- name or list of names of generators in model.
 
1586          eventTarget -- name or list of names of events in specified generator(s)
 
1587          val -- bisect limit (int > 0)
 
1588  
 
1589          val is applied to every listed event in every listed generator, if events and generators exist.
 
1590          """ 
1591          if ics is None: 
1592              ics = self.icdict 
1593          if isinstance(target, list): 
1594              for targName in target: 
1595                  estruct = self.modelInfo[targName]['dsi'].get('eventstruct', ics, t) 
1596                  estruct.setBisect(eventTarget, val) 
1597          else: 
1598              estruct = self.modelInfo[target]['dsi'].get('eventstruct', ics, t) 
1599              estruct.setBisect(eventTarget, val)

1600  
 


1601 -    def resetEventTimes(self):


1602          """Internal method""" 
1603          for ds in self.registry.values(): 
1604              ds.resetEventTimes()

1605  
 


1606 -    def _set_for_hybrid_DS(self, state):


1607          """Internal method to set all sub-models flag for being part of a hybrid
 
1608          trajectory computation.
 
1609          Useful to indicate that high level events should not be reset when a
 
1610          Generator is reused between hybrid trajectory segments.""" 
1611          for ds in self.registry.values(): 
1612              ds._set_for_hybrid_DS(state)

1613  
 


1614 -    def searchForNames(self, template):


1615          """Find parameter / variables names matching template in
 
1616          the model's generators or sub-models: the returned result is a list.""" 
1617          if self._mspecdict is None: 
1618              raise PyDSTool_ExistError("Cannot use this function for models"
 
1619                                        " not defined through ModelSpec") 
1620          result = {} 
1621          for modelName, mspecinfo in self._mspecdict.iteritems(): 
1622              # HACK to force compatibility of mspecinfo being a dict/args vs. a GDescriptor
 
1623              if isinstance(mspecinfo, dict): 
1624                  foundNames = searchModelSpec(mspecinfo['modelspec'], template) 
1625              else: 
1626                  # args or GDescriptor
 
1627                  foundNames = searchModelSpec(mspecinfo.modelspec, template) 
1628              result[modelName] = foundNames 
1629          return result

1630  
 
1631  
 


1632 -    def searchForVars(self, template):


1633          """Find variable and auxiliary variable names that have to be in every
 
1634          generator or sub-model: the returned result is a list.""" 
1635          if self._mspecdict is None: 
1636              raise PyDSTool_ExistError("Cannot use this function for models"
 
1637                                        " not defined through ModelSpec") 
1638          # all sub-models must define the same variables, so need only
 
1639          # to look at one
 
1640          a_ds_name = self._mspecdict.keys()[0] 
1641          a_ds_mspec = self._mspecdict[a_ds_name]['modelspec'] 
1642          foundNames = searchModelSpec(a_ds_mspec, template) 
1643          try: 
1644              fspec = self.registry[a_ds_name].get('funcspec') 
1645          except AttributeError: 
1646              # for non-hybrid models
 
1647              fspec = self.registry[a_ds_name].registry.values()[0].get('funcspec') 
1648          # funcspec won't have its internal names converted to
 
1649          # hierarchical form, so do it here on the attributes we
 
1650          # need
 
1651          return intersect(self.registry[a_ds_name]._FScompatibleNamesInv(fspec.vars + fspec.auxvars),
 
1652                               foundNames)

1653  
 
1654  
 
1655  
 
1656  
 


1657 -class NonHybridModel(Model):


1658 -    def __init__(self, *a, **kw):


1659          Model.__init__(self, True, *a, **kw) 
1660          # collect parameter info from all modelInfo objects
 
1661          self._generateParamInfo() 
1662          self._validateRegistry(self.obsvars, self.intvars)

1663  
 


1664 -    def _findTrajInitiator(self, end_reasons, partition_num, t0, xdict,
 
1665                                gi=None, swRules=None):


1666          # return GeneratorInterface object and include return
 
1667          # elements for compatibility with HybridModel._findTrajInitiator:
 
1668          # swRules, globalConRules, nextModelName, reused (True b/c always same),
 
1669          #       epochStateMaps, notDone
 
1670          infodict = self.modelInfo.values()[0] 
1671          return infodict['dsi'], infodict['swRules'], \
 
1672                 infodict['globalConRules'], infodict['dsi'].model.name, \
 
1673                 True, None, True

1674  
 


1675 -    def cleanupMemory(self):


1676          """Clean up memory usage from past runs of a solver that is interfaced through
 
1677          a dynamic link library. This will prevent the 'continue' integration option from
 
1678          being accessible and will delete other data about the last integration run.""" 
1679          self.registry.values()[0].cleanupMemory()

1680  
 


1681 -    def haveJacobian(self):


1682          """Returns True iff all objects in modelInfo have
 
1683          defined Jacobians.""" 
1684          return self.registry.values()[0].haveJacobian()

1685  
 


1686 -    def haveJacobian_pars(self):


1687          """Returns True iff all objects in modelInfo have
 
1688          defined Jacobians.""" 
1689          return self.registry.values()[0].haveJacobian_pars()

1690  
 


1691 -    def Rhs(self, t, xdict, pdict=None, asarray=False):


1692          """Direct access to a generator's Rhs function.
 
1693          Parameters:
 
1694  
 
1695            t        time (can use 0 for an autonomous system)
 
1696            xdict    state dictionary or Point.
 
1697            pdict    parameter dictionary or Point
 
1698                     (optional, default current parameters)
 
1699            asarray  (Bool, optional, default False) If true, will return an array
 
1700                     in state name alphabetical order, else a Point
 
1701          """ 
1702          # get Generator as 'ds'
 
1703          ds = self.registry.values()[0] 
1704          fscm = ds._FScompatibleNames 
1705          fscmInv = ds._FScompatibleNamesInv 
1706          vars = ds.get('funcspec').vars   # FS compatible 
1707          x_fs = filteredDict(fscm(xdict), vars) 
1708          # in case ds i.c.'s are different or not set yet (NaN's)
 
1709          # for purposes of auxiliary function 'initcond' calls during Rhs
 
1710          old_ics = ds.initialconditions.copy() 
1711          ds.initialconditions.update(x_fs) 
1712          x_fs.update(ds.initialconditions) 
1713          # don't need to convert pdict names to FS-compatible as they sort
 
1714          # the same
 
1715          if pdict is None: 
1716              pdict = self.pars 
1717          if asarray: 
1718              rhsval = array(ds.Rhs(t, x_fs, pdict)) 
1719          else: 
1720              rhsval = Point({'coorddict': dict(zip(fscmInv(vars),
 
1721                                                ds.Rhs(t, x_fs, pdict))),
 
1722                        'coordtype': float,
 
1723                        'norm': self._normord}) 
1724          ds.initialconditions.update(old_ics) 
1725          return rhsval

1726  
 


1727 -    def Jacobian(self, t, xdict, pdict=None, asarray=False):


1728          """Direct access to a generator's Jacobian function (if defined).
 
1729  
 
1730          Arguments:
 
1731  
 
1732            t        time (can use 0 for an autonomous system)
 
1733            xdict    state dictionary or Point.
 
1734            pdict    parameter dictionary or Point
 
1735                     (optional, default current parameters)
 
1736            asarray  (Bool, optional, default False) If true, will return an array
 
1737                     in state name alphabetical order, else a Point
 
1738          """ 
1739          ds = self.registry.values()[0] 
1740          if not ds.haveJacobian(): 
1741              raise PyDSTool_ExistError("Jacobian not defined") 
1742          fscm = ds._FScompatibleNames 
1743          fscmInv = ds._FScompatibleNamesInv 
1744          vars = ds.get('funcspec').vars   # FS compatible 
1745          x_fs = filteredDict(fscm(xdict), vars) 
1746          # in case ds i.c.'s are different or not set yet (NaN's)
 
1747          # for purposes of auxiliary function 'initcond' calls during Rhs
 
1748          old_ics = ds.initialconditions.copy() 
1749          ds.initialconditions.update(x_fs) 
1750          x_fs.update(ds.initialconditions) 
1751          # don't need to convert pdict names to FS-compatible as they sort
 
1752          # the same
 
1753          if pdict is None: 
1754              pdict = self.pars 
1755          if asarray: 
1756              J = array(ds.Jacobian(t, x_fs, pdict)) 
1757          else: 
1758              J = Pointset({'coorddict': dict(zip(fscmInv(vars),
 
1759                                           ds.Jacobian(t, x_fs, pdict))),
 
1760                           'coordtype': float,
 
1761                           'norm': self._normord}) 
1762          ds.initialconditions.update(old_ics) 
1763          return J

1764  
 


1765 -    def JacobianP(self, t, xdict, pdict=None, asarray=False):


1766          """Direct access to a generator's JacobianP function (if defined).
 
1767  
 
1768          Arguments:
 
1769  
 
1770            t        time (can use 0 for an autonomous system)
 
1771            xdict    state dictionary or Point.
 
1772            pdict    parameter dictionary or Point
 
1773                     (optional, default current parameters)
 
1774            asarray  (Bool, optional, default False) If true, will return an array
 
1775                     in state name alphabetical order, else a Point
 
1776          """ 
1777          ds = self.registry.values()[0] 
1778          if not ds.haveJacobian_pars(): 
1779              raise PyDSTool_ExistError("Jacobian w.r.t. pars not defined") 
1780          fscm = ds._FScompatibleNames 
1781          fscmInv = ds._FScompatibleNamesInv 
1782          x_fs = filteredDict(fscm(xdict), ds.get('funcspec').vars) 
1783          # in case ds i.c.'s are different or not set yet (NaN's)
 
1784          # for purposes of auxiliary function 'initcond' calls during Rhs
 
1785          old_ics = ds.initialconditions.copy() 
1786          ds.initialconditions.update(x_fs) 
1787          x_fs.update(ds.initialconditions) 
1788          # don't need to convert pdict names to FS-compatible as they sort
 
1789          # the same
 
1790          if pdict is None: 
1791              pdict = self.pars 
1792          if asarray: 
1793              Jp = array(ds.JacobianP(t, x_fs, pdict)) 
1794          else: 
1795              Jp = Pointset({'coorddict': dict(zip(fscmInv(ds.get('funcspec').pars),
 
1796                                            ds.JacobianP(t, x_fs, pdict))),
 
1797                           'coordtype': float,
 
1798                           'norm': self._normord}) 
1799          ds.initialconditions.update(old_ics) 
1800          return Jp

1801  
 


1802 -    def MassMatrix(self, t, xdict, pdict=None, asarray=False):


1803          """Direct access to a generator's MassMatrix function (if defined).
 
1804  
 
1805          Arguments:
 
1806  
 
1807            t        time (can use 0 for an autonomous system)
 
1808            xdict    state dictionary or Point.
 
1809            pdict    parameter dictionary or Point
 
1810                     (optional, default current parameters)
 
1811            asarray  (Bool, optional, default False) If true, will return an array
 
1812                     in state name alphabetical order, else a Point
 
1813          """ 
1814          ds = self.registry.values()[0] 
1815          if not ds.haveMass(): 
1816              raise PyDSTool_ExistError("Mass matrix not defined") 
1817          fscm = ds._FScompatibleNames 
1818          fscmInv = ds._FScompatibleNamesInv 
1819          vars = ds.get('funcspec').vars   # FS compatible 
1820          x_fs = filteredDict(fscm(xdict), vars) 
1821          # in case ds i.c.'s are different or not set yet (NaN's)
 
1822          # for purposes of auxiliary function 'initcond' calls during Rhs
 
1823          old_ics = ds.initialconditions.copy() 
1824          ds.initialconditions.update(x_fs) 
1825          x_fs.update(ds.initialconditions) 
1826          # don't need to convert pdict names to FS-compatible as they sort
 
1827          # the same
 
1828          if pdict is None: 
1829              pdict = self.pars 
1830          if asarray: 
1831              M = array(ds.MassMatrix(t, x_fs, pdict)) 
1832          else: 
1833              M = Point({'coorddict': dict(zip(fscmInv(vars),
 
1834                                          ds.MassMatrix(t, x_fs, pdict))),
 
1835                        'coordtype': float,
 
1836                        'norm': self._normord}) 
1837          ds.initialconditions.update(old_ics) 
1838          return M

1839  
 


1840 -    def AuxVars(self, t, xdict, pdict=None, asarray=False):


1841          """Direct access to a generator's auxiliary variables
 
1842          definition (if defined).
 
1843  
 
1844          Arguments:
 
1845  
 
1846            t        time (can use 0 for an autonomous system)
 
1847            xdict    state dictionary or Point.
 
1848            pdict    parameter dictionary or Point
 
1849                     (optional, default current parameters)
 
1850            asarray  (Bool, optional, default False) If true, will return an array
 
1851                     in state name alphabetical order, else a Point
 
1852          """ 
1853          fscm = ds._FScompatibleNames 
1854          fscmInv = ds._FScompatibleNamesInv 
1855          x_fs = filteredDict(fscm(xdict), ds.get('funcspec').vars) 
1856          # in case ds i.c.'s are different or not set yet (NaN's)
 
1857          # for purposes of auxiliary function 'initcond' calls during Rhs
 
1858          old_ics = ds.initialconditions.copy() 
1859          ds.initialconditions.update(x_fs) 
1860          x_fs.update(ds.initialconditions) 
1861          # don't need to convert pdict names to FS-compatible as they sort
 
1862          # the same
 
1863          if pdict is None: 
1864              pdict = self.pars 
1865          if asarray: 
1866              A = array(ds.AuxVars(t, x_fs, pdict)) 
1867          else: 
1868              A = Point({'coorddict': dict(zip(fscmInv(ds.get('funcspec').auxvars),
 
1869                                  ds.AuxVars(t, x_fs, pdict))),
 
1870                        'coordtype': float,
 
1871                        'norm': self._normord}) 
1872          ds.initialconditions.update(old_ics) 
1873          return A

1874  
 
1875  
 


1876 -    def compute(self, trajname, **kw):


1877          """Compute a non-hybrid trajectory. Returns a Trajectory object.
 
1878  
 
1879          Arguments (non-keyword):
 
1880            trajname   Name of trajectory to create (string)
 
1881  
 
1882          Arguments (keyword only, all optional):
 
1883            force      (Bool, default False) - force overwrite of any trajectory
 
1884                       stored in this object with the same name
 
1885            verboselevel  (int, default 0)
 
1886            ics        initial conditions dict or Point
 
1887            pars       parameters dict or Point
 
1888            tdata      time data (interval as sequence of 2 numeric values)
 
1889          """ 
1890          tdata, t0_global, t1_global, force_overwrite = \
 
1891                  Model._prepareCompute(self, trajname, **kw) 
1892          if self.icdict == {}: 
1893              # just get i.c. from the single generator,
 
1894              # making sure it's non-empty
 
1895              self.icdict = self.registry.values()[0].get('initialconditions') 
1896              if self.icdict == {}: 
1897                  # gen's i.c.s were empty too
 
1898                  raise PyDSTool_ExistError("No initial conditions specified") 
1899          xdict = {} 
1900          for xname, value in self.icdict.iteritems(): 
1901              #if xname not in self.obsvars+self.intvars:
 
1902              #    raise ValueError("Invalid variable name in initial "
 
1903              #                       "conditions: " + xname)
 
1904              xdict[xname] = ensurefloat(value) 
1905          # clean up self.icdict
 
1906          self.icdict = xdict.copy() 
1907  
 
1908          ## compute trajectory segment until an event or t1 is reached
 
1909          gen = self.registry.values()[0]     # sole entry, a Generator 
1910  #        print "N-h model before fiddling: t0_global =", t0_global, "t1_global =", t1_global
 
1911          t0_global += gen.globalt0 
1912          t1_global += gen.globalt0 
1913          t0 = t0_global    # initial value 
1914          if t1_global > gen.indepvariable.indepdomain[1]+t0: 
1915              if isfinite(gen.indepvariable.indepdomain[1]): 
1916                  if self.verboselevel > 0: 
1917                      print "Warning: end time was truncated to max size of specified independent variable domain" 
1918                  t1 = gen.indepvariable.indepdomain[1]+t0 
1919              else: 
1920                  t1 = t1_global 
1921          else: 
1922              t1 = t1_global 
1923  
 
1924          # For sub-models that support setPars() and
 
1925          # compute() methods, set the sub-model's global time
 
1926          # reference (in case it needs it), and verify that t=0 is valid
 
1927          # in the sub-model's time domain
 
1928  #        print "\nNonhybrid model %s 'compute'"%self.name
 
1929  #        print "  generator global t0 =", gen.globalt0
 
1930  #        print "  model t0_global=", t0_global, "t1_global=",t1_global
 
1931  #        print "  original model tdata = [%f,%f], gen tdata = [0,%f]"%(tdata[0],tdata[1],t1-t0), " and globalt0=",t0
 
1932  
 
1933          # add remaining pars for system
 
1934          setup_pars = {'ics': xdict, 'tdata': [0, t1-t0]} 
1935          if self._abseps is not None: 
1936              setup_pars['abseps'] = self._abseps 
1937          if hasattr(gen, 'algparams'): 
1938              setup_pars['algparams'] = {'verbose': self.verboselevel} 
1939              try: 
1940                  if gen.algparams['init_step'] > t1-t0: 
1941                      if self.verboselevel > 0: 
1942                          print "Warning: time step too large for remaining time"\
 
1943                                + " interval. Temporarily reducing time step to " \
 
1944                                + "1/10th of its previous value" 
1945                      setup_pars['algparams'].update({'init_step': (t1-t0)/10}) 
1946              except (AttributeError, KeyError): 
1947                  # system does not support this integration parameter
 
1948                  pass 
1949          # if external inputs used, make sure any parameter-bound
 
1950          # global t0 offsets are updated in case epoch state map changed them
 
1951          if self._inputt0_par_links != {}: 
1952              t0val_dict = {} 
1953              for inp, val in self._inputt0_par_links.items(): 
1954                  t0val_dict[inp] = self.pars[val] 
1955              gen.set(inputs_t0=t0val_dict) 
1956          gen.set(**setup_pars) 
1957          gen.diagnostics.clearWarnings() 
1958          gen.diagnostics.clearErrors() 
1959  
 
1960          #### Compute a trajectory segment as a NonHybrid Trajectory
 
1961          #print "Entering generator %s at t0=%f, t1=%f"%(gen.name, t0, t1)
 
1962          try: 
1963              traj = gen.compute(trajname+'_0') 
1964          except PyDSTool_ValueError, e: 
1965              print "\nError in Generator:", gen.name 
1966              gen.diagnostics.showWarnings() 
1967              gen.diagnostics.showErrors() 
1968              print "Are the constituent generator absolute epsilon " 
1969              print " tolerances too small? -- this abseps =", gen._abseps 
1970              raise 
1971          except KeyboardInterrupt: 
1972              raise 
1973          except: 
1974              print "\nError in Generator:", gen.name 
1975              gen.diagnostics.showWarnings() 
1976              gen.diagnostics.showErrors() 
1977              self.diagnostics.traceback = {} 
1978              for k,v in gen.diagnostics.traceback.iteritems(): 
1979                  if isinstance(v, dict): 
1980                      dentry = {} 
1981                      for vk, vv in v.iteritems(): 
1982                          dentry[gen._FScompatibleNamesInv(vk)] = vv 
1983                  else: 
1984                      dentry = v 
1985                  self.diagnostics.traceback[k] = dentry 
1986              if self.diagnostics.traceback != {}: 
1987                  print "Traceback dictionary copied to model" 
1988              raise 
1989          if traj is None: 
1990              raise ValueError("Generator %s failed to create a trajectory"%gen.name) 
1991          else: 
1992              if self._abseps is not None: 
1993                  assert traj.indepdomain._abseps == self._abseps 
1994                  for v in traj.depdomain.values(): 
1995                      assert v._abseps == self._abseps 
1996          if not isparameterized(traj): 
1997              raise ValueError("Generator " + gen.name + " produced a " \
 
1998                               + " non-parameterized Trajectory") 
1999          # update original copy of the generator with new event times during
 
2000          # this trajectory, for high level event detection to be able to check
 
2001          # eventinterval for terminal events between hybrid steps
 
2002          # ?????
 
2003          ##self.modelInfo[gen.name]['dsi'].eventstruct = copy.copy(gen.eventstruct)
 
2004  
 
2005          #### Post-process trajectory segment:
 
2006          # look at warnings etc. for any terminating events that occurred
 
2007          if gen.diagnostics.hasErrors() and self.verboselevel > 0: 
2008              for e in gen.diagnostics.errors: 
2009                  print 'Generator ' + gen.name + \
 
2010                                      ' in trajectory segment had errors' 
2011                  gen.diagnostics.showErrors() 
2012          end_reasons = ['time'] 
2013          # NB. end reason will always be 'time' for ExplicitFnGen
 
2014          # Default time interval after Generator completed,
 
2015          # in case it was truncated by a terminal event
 
2016          time_interval = traj.indepdomain 
2017          ti_1 = time_interval[1] 
2018          if gen.diagnostics.hasWarnings(): 
2019              if self.verboselevel >= 1: 
2020                  gen.diagnostics.showWarnings() 
2021              for w in gen.diagnostics.warnings: 
2022                  if w[0] == Generator.W_TERMEVENT or \
 
2023                     w[0] == Generator.W_TERMSTATEBD: 
2024                      if self.verboselevel > 1: 
2025                          print "Time:", ti_1+t0 
2026                          print 'Generator ' + gen.name + \
 
2027                              ' had a terminal event. Details (in local ' + \
 
2028                              'system time) ...\n ' + str(w[1]) 
2029                      # w[1] always has t as first entry, and
 
2030                      # either a state variable name
 
2031                      # or a list of terminal event names as second.
 
2032                      if isinstance(w[1][1], list): 
2033                          if len(w[1][1]) > 1: 
2034                              if self.verboselevel > 0: 
2035                                  print 'Warning: More than one terminal event found.' 
2036                                  print '  Consider altering event pars.' 
2037                          end_reasons = [w[1][1][ri] \
 
2038                                 for ri in range(len(w[1][1]))] 
2039                      else: 
2040                          end_reasons = [w[1][1]] 
2041                      #print "End reason: ", end_reasons[0]
 
2042              self.diagnostics.update(gen.diagnostics) 
2043  
 
2044          # Apply global consistency conditions, and truncate
 
2045          # trajectory to the last OK index in the independent variable
 
2046          # if any fail, for those conditions that do not apply to the
 
2047          # whole trajectory in an accept/reject fashion. If any fail
 
2048          # without specifying an index then the whole trajectory is being
 
2049          # rejected.
 
2050          global_end_reasons = {} 
2051          global_end_ixs = [] 
2052          dsi = self.modelInfo.values()[0]['dsi'] 
2053          for globalDS in self.modelInfo.values()[0]['globalConRules']: 
2054              if globalDS(dsi): 
2055                  global_end_reasons[Inf] = \
 
2056                          globalDS.conditions.collate_results('reasons',
 
2057                                                          merge_lists=True) 
2058                  global_end_ixs.append(Inf) 
2059              else: 
2060                  # if global consistency fails then the features *must* provide
 
2061                  # the last OK position in the trajectory to truncate to,
 
2062                  # otherwise the whole trajectory FAILs.
 
2063                  try: 
2064                      final_ok_idx = globalDS.conditions._find_idx() 
2065                  except (AttributeError, RuntimeError): 
2066                      print "Trajectory creation failed..." 
2067                      globalDS.conditions.results.info() 
2068                      raise PyDSTool_ExistError("Global consistency checks failed for"
 
2069                              " model interface %s for trajectory %s"%(str(globalDS),
 
2070                                                                   traj.name)) 
2071                  else: 
2072                      if final_ok_idx is not None: 
2073                          global_end_ixs.append(final_ok_idx) 
2074                      global_end_reasons[final_ok_idx] = \
 
2075                              globalDS.conditions.collate_results('reasons',
 
2076                                                          merge_lists=True) 
2077          if len(global_end_reasons) > 0: 
2078              smallest_ok_idx = min(global_end_ixs) 
2079              # this idx may be Inf if globalDS passed all points OK
 
2080              # - but it may have still changed (refined) the end reason
 
2081              if isfinite(smallest_ok_idx): 
2082                  # truncate in place
 
2083                  traj.truncate_to_idx(smallest_ok_idx) 
2084              # overwrite current end reason
 
2085              end_reasons = global_end_reasons[smallest_ok_idx] 
2086          if self.verboselevel > 1: 
2087              print "End reason in %s was %s"%(self.name,str(end_reasons)) 
2088          # DEBUG
 
2089  #        print "Nonhybrid traj info:", traj.indepdomain.get(), traj.globalt0, type(traj)
 
2090  #        print "traj vars:", traj.variables.keys(), "\n"
 
2091  
 
2092          #### Clean up
 
2093          if ti_1 > t1_global-t0: 
2094              print "Warning: Generator time interval exceeds prescribed limits:" 
2095              print " ... ", ti_1, " > ", t1_global-t0 
2096          epochEvents = {} 
2097          try: 
2098              epochEvents.update(gen.getEvents(asGlobalTime=True)) 
2099          except (AttributeError, TypeError): 
2100              # this Generator has no eventstruct
 
2101              pass 
2102  
 
2103          ## New code in Trajectory.__init__ takes care of this now
 
2104          # Take a copy of the events as they were at the time that the
 
2105          # trajectory was computed, for future reference
 
2106          #traj.events = epochEvents
 
2107          #traj._createEventTimes()
 
2108          # TEMP
 
2109          #print "Nonhybridmodel - temp removal of eventstruct non-copy"
 
2110          #traj.modelEventStructs = gen.eventstruct #copy.deepcopy(gen.eventstruct)
 
2111          self.trajectories[trajname] = traj

2112  
 
2113  
 


2114 -    def _validateRegistry(self, obsvars, intvars):


2115          """Validate Model's modelInfo attribute.""" 
2116          # ensure that modelInfo is a single Generator object only
 
2117          assert len(self.modelInfo) == 1, \
 
2118                 "Non-hybrid model must contain a single Generator" 
2119          infodict = self.modelInfo.values()[0] 
2120          if not isinstance(infodict['dsi'], MProject.GeneratorInterface): 
2121              raise TypeError("Must provide a single Generator object"
 
2122                              " wrapped in a GeneratorInterface")

2123  
 


2124 -    def _infostr(self, verbose=1):


2125          if verbose > 0: 
2126              outputStr = 'Non-Hybrid Model '+self.name+" containing components:" 
2127              print "Observable variables:", self.obsvars 
2128              print "Internal variables:", self.intvars 
2129              print "Auxiliary variables:", self.auxvars 
2130              name, infodict = self.modelInfo.items()[0] 
2131              outputStr += "\n--- Generator: "+name 
2132              outputStr += "\n  "+infodict['dsi'].model._infostr(verbose-1) 
2133          else: 
2134              outputStr = 'Non-Hybrid Model '+self.name 
2135          return outputStr

2136  
 
2137  
 
2138  
 


2139 -class HybridModel(Model):


2140      """
 
2141      obsvars specifies the observable
 
2142      variables for this model, which must be present in all
 
2143      trajectory segments (regardless of which other variables are
 
2144      specified in those segments). intvars specifies
 
2145      non-observable (internal) variables for the model, which
 
2146      may or may not be present in trajectory segments, depending
 
2147      on reductions, etc., that are present in the sub-model (e.g.
 
2148      generator) that determines the trajectory segment.
 
2149      """ 


2150 -    def __init__(self, *a, **kw):


2151          Model.__init__(self, True, *a, **kw) 
2152          # collect parameter info from all modelInfo objects
 
2153          self._generateParamInfo() 
2154          # Ensure all Generators provided to build trajectory share the same
 
2155          # observables, and that modelInfo switch rules mappings are OK
 
2156          self._validateRegistry(self.obsvars, self.intvars) 
2157          # Check that all pars of the same name are equal
 
2158          self._validateParameters()

2159  
 


2160 -    def _prepareICs(self, xdict, traj, ds, t0, ti_1):


2161          # ds_old no longer needed
 
2162          # get new initial conditions for this epoch (partition)
 
2163          # DEBUG
 
2164  #        print "\n\n_prepareICs for HybridModel %s and old traj %s"%(self.name,traj.name)
 
2165  #        print "t0=%f, ti_1=%f"%(t0,ti_1)
 
2166  #        print "old traj globalt0 =", traj.globalt0
 
2167  #        print "t domain =", traj.indepdomain.get()
 
2168  #        print dict(traj(traj.indepdomain[1], asGlobalTime=False)), "\n"
 
2169          for xname in self.allvars+self.auxvars: 
2170              # update final condition of epoch to be used to generate
 
2171              # next initial condition when we re-enter the loop
 
2172              xname_compat = traj._FScompatibleNames(xname) 
2173              try: 
2174                  xdict[xname] = traj.variables[xname_compat].output.datapoints[1][-1] 
2175              except KeyError: 
2176                  # e.g. auxiliary var didn't need calling
 
2177                  pass 
2178              except AttributeError: 
2179                  # non-point based output attributes of a Variable need
 
2180                  # to be 'called' ...
 
2181                  try: 
2182                      xdict[xname] = traj.variables[xname_compat](ti_1) 
2183                  except RuntimeError: 
2184                      # traj is a hybrid traj
 
2185                      print "**********" 
2186                      print xname, " dep domain:", traj.variables[xname_compat].depdomain.get() 
2187                      print traj.depdomain[xname_compat].get() 
2188                      print "indep domain:", traj.variables[xname_compat].indepdomain.get() 
2189                      print "Var val at t-eps %f = "%(ti_1-1e-4), \
 
2190                             traj.variables[xname_compat](ti_1-1e-4) 
2191                      raise 
2192                  except: 
2193                      print "**********" 
2194                      print xname, " dep domain:", traj.variables[xname_compat].depdomain.get() 
2195                      print traj.depdomain[xname_compat].get() 
2196                      print traj.trajSeq[-1].variables[xname_compat].depdomain.get() 
2197                      raise 
2198              except PyDSTool_BoundsError: 
2199                  print "Value out of bounds in variable call:" 
2200                  print "  variable '%s' was called at time %f"%(xname, ti_1) 
2201                  raise

2202  
 
2203  
 


2204 -    def _applyStateMap(self, epochStateMaps, model_interface,
 
2205                         next_model_interface, traj, xdict, t0):


2206          # the mapping might be the ID fn
 
2207          num_maps = len(epochStateMaps) 
2208          # apply all maps to the initial condition
 
2209          # but check that the ordering doesn't matter (they are
 
2210          # commutative), otherwise tell the user we can't safely
 
2211          # proceed [FOR NOW, JUST DON'T ALLOW SIMULTANEOUS EVENTS]
 
2212          if num_maps > 1: 
2213              # ensure that all the maps are the same for multiple
 
2214              # simultaneous events that point to the same generator
 
2215              # (the latter was already verified above)
 
2216              for mapix in range(1,num_maps): 
2217                  if epochStateMaps[0] != epochStateMaps[mapix]: 
2218                      raise RuntimeError("PyDSTool does not yet allow "
 
2219                           "truly simultaneous events that do not point "
 
2220                           "to the same model with the same mapping") 
2221          # Now work out new inputs state
 
2222          dsinps = model_interface.get('inputs', xdict, t0) 
2223          if dsinps != {}: 
2224              # build dictionary of initial values for the
 
2225              # external inputs
 
2226              extinputs_ic = {}.fromkeys(dsinps) 
2227              try: 
2228                  for k in extinputs_ic: 
2229                      extinputs_ic[k] = dsinps[k](t0) 
2230              except PyDSTool_BoundsError: 
2231                  print "Cannot proceed - sub-model '" + model_interface.model.name \
 
2232                        + "' had an external input undefined " \
 
2233                        + "at t =", t0 
2234                  raise 
2235          else: 
2236              extinputs_ic = {} 
2237          eventstruct = model_interface.get('eventstruct', xdict, t0) 
2238          pars = model_interface.get('pars', xdict, t0) 
2239          pars_temp = traj._FScompatibleNames(pars) 
2240          xdict_temp = traj._FScompatibleNames(xdict) 
2241          for epochStateMap in epochStateMaps: 
2242              # epochStateMap might update xdict_temp and pars_dict
 
2243              epochStateMap.evmapping(xdict_temp, pars_temp,
 
2244                              extinputs_ic, eventstruct, t0) 
2245          # use update method to change dicts *in place*
 
2246          pars.update(traj._FScompatibleNamesInv(pars_temp)) 
2247          # update next MI's parameters if mapped by the event mapping
 
2248          # (the one corresponding to the original xdict)
 
2249          try: 
2250              next_model_interface.set('pars', pars, xdict, t0) 
2251          except (PyDSTool_AttributeError, PyDSTool_ValueError): 
2252              # next model may not have these params, so just ignore
 
2253              pass 
2254          xdict.update(traj._FScompatibleNamesInv(xdict_temp)) 
2255          # No longer need the aux vars
 
2256          for xname in self.auxvars: 
2257              try: 
2258                  del xdict[xname] 
2259              except KeyError: 
2260                  # aux var value never put here
 
2261                  pass

2262  
 


2263 -    def cleanupMemory(self):


2264          """Clean up memory usage from past runs of a solver that is interfaced
 
2265          through a dynamic link library. This will prevent the 'continue'
 
2266          integration option from being accessible and will delete other data
 
2267          about the last integration run.
 
2268          """ 
2269          for MI in self.registry.values(): 
2270              MI.model.cleanupMemory()

2271  
 


2272 -    def _findTrajInitiator(self, end_reasons, partition_num, t0, xdict,
 
2273                                mi=None, swRules=None):


2274          # Initial values for these
 
2275          epochStateMaps = None 
2276          notDone = True 
2277          reused = False 
2278          if end_reasons is None: 
2279              # first time in the while loop, so set up xnames
 
2280              assert partition_num == 0, ("end_reasons was None on a "
 
2281                                          "non-initial epoch") 
2282              xnames = self.icdict.keys() 
2283              xnames.sort() 
2284              # only for the initial Generator of a trajectory
 
2285              try: 
2286                  infodict = findTrajInitiator(self.modelInfo, t0, xdict,
 
2287                                          self.pars, self.intvars,
 
2288                                          self.verboselevel) 
2289              except PyDSTool_ValueError, errinfo: 
2290                  print errinfo 
2291                  raise PyDSTool_ExistError('No unique eligible Model found:'
 
2292                    ' cannot continue (check active terminal event definitions'
 
2293                    ' or error message above)') 
2294              mi = infodict['dsi'] 
2295              swRules = infodict['swRules'] 
2296              globalConRules = infodict['globalConRules'] 
2297              missing = remain(mi.get('variables', ics=xdict, t0=t0).keys(),
 
2298                            xnames+self.auxvars+self.intvars) 
2299              if missing != []: 
2300                  raise AssertionError('Missing initial condition specifications'
 
2301                                       ' for %s' % str(missing)) 
2302              for entry, val in xdict.iteritems(): 
2303                  if not isfinite(val): 
2304                      print "Warning: %s initial condition for "%mi.model.name \
 
2305                             + str(entry) + " = " + str(val) 
2306              if self.verboselevel > 1: 
2307                  print "\nStarting partition 0" 
2308                  print "Chose initiator '%s'"%mi.model.name 
2309              nextModelName = None 
2310          else: 
2311              # find new Generator using switching rules
 
2312              num_reasons = len(end_reasons) 
2313              # num_reasons > 1 will occur most often
 
2314              # for discrete time systems
 
2315              if num_reasons > 1: 
2316                  # then all next generators must be the same!
 
2317                  candidateNextModel = "" 
2318                  mapinfos = [] 
2319                  for ri in range(num_reasons): 
2320                      try: 
2321                          mapinfos.append(swRules[end_reasons[ri]]) 
2322                      except KeyError: 
2323                          # no rule for this termination reason
 
2324                          continue 
2325                      nextModelName = mapinfos[-1][0] 
2326                      if candidateNextModel == "": 
2327                          candidateNextModel = nextModelName 
2328                      else: 
2329                          if candidateNextModel != nextModelName: 
2330                              raise RuntimeError("The multiple reasons "
 
2331                                "for terminating last trajectory segment"
 
2332                                " did not point to the same generator to"
 
2333                                " continue the calculation.") 
2334                  if len(mapinfos) == 0: 
2335                      # No next generators found
 
2336                      nextModelName = 'terminate' 
2337                  else: 
2338                      epochStateMaps = [m[1] for m in mapinfos] 
2339              else: 
2340                  if end_reasons[0] in swRules: 
2341                      mapinfo = swRules[end_reasons[0]] 
2342                      nextModelName = mapinfo[0] 
2343                      epochStateMaps = [mapinfo[1]] 
2344                  else: 
2345                      nextModelName = 'terminate' 
2346                      epochStateMaps = None   # will not be needed 
2347              if nextModelName == 'terminate': 
2348                  if self.verboselevel > 0: 
2349                      print 'Trajectory calculation for Model `'+mi.model.name\
 
2350                            +'` terminated without a DS specified in switch'\
 
2351                            +' rules with which to continue.' 
2352                  globalConRules = None 
2353                  notDone = False   # terminates compute method 
2354              elif nextModelName == mi.model.name: 
2355                  # Reuse the same model copy (take advantage of
 
2356                  # not having to re-copy the model from modelInfo)
 
2357                  # Currently we can't use the continue integration
 
2358                  # option of ODE solvers because of the way that each
 
2359                  # new hybrid partition is solved in "local time", i.e.
 
2360                  # from t=0. So this would mess up the solvers`
 
2361                  # 'continue' option.
 
2362                  reused = True   # not used in compute 
2363                  # swRules is untouched
 
2364                  # return same globalConRules as had before
 
2365                  globalConRules = self.modelInfo[nextModelName]['globalConRules'] 
2366              else: 
2367                  next = self.modelInfo[nextModelName] 
2368                  mi = next['dsi'] 
2369                  swRules = next['swRules'] 
2370                  globalConRules = next['globalConRules'] 
2371          if self.verboselevel > 1: 
2372              print "\nStarting partition #%i"%partition_num 
2373              print "Chose model '%s'"%mi.model.name 
2374          return mi, swRules, globalConRules, nextModelName, reused, \
 
2375                 epochStateMaps, notDone

2376  
 


2377 -    def _addTraj(self, trajname, trajseq, epochEvents,
 
2378                   modelNames, force_overwrite=False):


2379          """Add a computed trajectory to database.""" 
2380  
 
2381          if not force_overwrite: 
2382              assert trajname not in self.trajectories, \
 
2383                     'Trajectory name already used' 
2384          #genEvStructs = dict(zip(modelNames,
 
2385          #                [copy.copy(self.modelInfo[gn]['dsi'].eventstruct) \
 
2386          #                           for gn in modelNames]))
 
2387          # TEMP
 
2388          #print "Model: avoid copying eventstructs?"
 
2389          genEvStructs = dict(zip(modelNames,
 
2390                          [self.modelInfo[gn]['dsi'].eventstruct \
 
2391                                     for gn in modelNames])) 
2392          # time_range is whole time range over sequence
 
2393          time_range = [None, None] 
2394          indepvarname = '' 
2395          genEventTimes = {}   # keyed by event name 
2396          genEvents = {}    # keyed by event name 
2397          # collate all FScompatible name maps from trajectories
 
2398          FScompatibleNames = symbolMapClass() 
2399          FScompatibleNamesInv = symbolMapClass() 
2400          for traj_ix, traj in enumerate(trajseq): 
2401              assert isinstance(traj, Trajectory), \
 
2402                     ('traj must contain trajectories') 
2403              if indepvarname == '': 
2404                  indepvarname = traj.indepvarname 
2405  ##            else:
 
2406  ##                if indepvarname != traj.indepvarname:
 
2407  ##                    print indepvarname, traj.indepvarname
 
2408  ##                    raise ValueError("Independent "
 
2409  ##                                        "variable name mismatch in trajectory")
 
2410              FScompatibleNames.update(traj._FScompatibleNames) 
2411              FScompatibleNamesInv.update(traj._FScompatibleNamesInv) 
2412              for evname in epochEvents[traj_ix]: 
2413                  if evname in genEvents and genEvents[evname] is not None: 
2414                      val = mapNames(traj._FScompatibleNamesInv,
 
2415                                         epochEvents[traj_ix][evname]) 
2416                      if val is not None: 
2417                          try: 
2418                              genEvents[evname].append(val) 
2419                          except ValueError: 
2420                              # in extreme cases with highest precision events,
 
2421                              # may have an "equal" time being added
 
2422                              if genEventTimes[evname][-1] - val['t'][0] > 0: 
2423                                  # some other ValueError
 
2424                                  raise 
2425                              else: 
2426                                  # don't add this point, it's identical
 
2427                                  pass 
2428                          else: 
2429                              try: 
2430                                  genEventTimes[evname].extend( \
 
2431                                      val.indepvararray.tolist()) 
2432                              except AttributeError: 
2433                                  pass 
2434                  else: 
2435                      genEvents[evname] = mapNames(traj._FScompatibleNamesInv,
 
2436                                            epochEvents[traj_ix][evname]) 
2437                      try: 
2438                          genEventTimes[evname] = \
 
2439                              epochEvents[traj_ix][evname].indepvararray.tolist() 
2440                      except AttributeError: 
2441                          pass 
2442              if time_range[0] is None: 
2443                  # first partition of sequence
 
2444                  DS_t_numtype = traj.indepdomain.type 
2445                  if isinputcts(traj): 
2446                      DS_t_typestr = 'continuous' 
2447                      # continuous time must be float
 
2448                      if not compareNumTypes(DS_t_numtype, _all_float): 
2449                          raise TypeError('continuous time inconsistent with '
 
2450                                          'non-float type') 
2451                  else: 
2452                      DS_t_typestr = 'discrete' 
2453                      # discrete time can be floats or ints
 
2454                  traj_vars = traj._FScompatibleNamesInv(traj.variables) 
2455                  assert intersect(self.allvars, traj_vars.keys()), \
 
2456                     "variable name for traj not present in sub-model's variables" 
2457                  # Pick one observable variable ('varname') to test with -- they
 
2458                  # must all have the same type. This variable must be present in
 
2459                  # all trajectory segments.
 
2460                  varname = self.obsvars[0] 
2461                  if varname in traj_vars: 
2462                      DS_x_numtype = traj_vars[varname].depdomain.type 
2463                  else: 
2464                      raise ValueError("varname not known") 
2465  ##                assert DS_x_numtype == self.obsvars[varname], \
 
2466  ##                       ('Mismatch between declared type of variable and '
 
2467  ##                        'that found in trajectory segment')
 
2468                  time_range = traj.indepdomain.get() 
2469                  try: 
2470                      time_range[0] += traj.globalt0 
2471                      time_range[1] += traj.globalt0 
2472                  except IndexError: 
2473                      raise ValueError('time interval of segment in hybrid '
 
2474                                              'trajectory cannot be singleton') 
2475                  # DEBUG
 
2476                  # temp
 
2477  #                print "\n---- %s _addTraj case 1: "%self.name
 
2478  #                print traj.indepdomain.get()
 
2479  #                print time_range
 
2480  #                print traj.globalt0
 
2481                  time_partitions = [(traj.indepdomain, \
 
2482                                      traj.globalt0, \
 
2483                                      traj.checklevel)]  # initial value 
2484              else: 
2485                  # remove the following line to support trajectories that
 
2486                  # only have partially-defined variable information, esp.
 
2487                  # if part is a map (vars defined only at discrete times)
 
2488                  if not compareNumTypes(DS_t_numtype, traj.indepdomain.type): 
2489                      raise TypeError('Mismatched time types for hybrid '
 
2490                                      'trajectory sequence') 
2491  #                print "\n---- %s _addTraj case 2: "%self.name
 
2492  #                print traj.indepdomain.get()
 
2493  #                temp = copy.copy(time_range)
 
2494  #                temp[1] += traj.indepdomain[1]
 
2495  #                print temp
 
2496  #                print traj.globalt0
 
2497                  if not traj.indepdomain.atEndPoint(time_range[1] \
 
2498                                                      - traj.globalt0, 'lo'): 
2499                      # temp
 
2500  #                    print "\n***", time_range
 
2501  #                    print traj.indepdomain.get()
 
2502  #                    print traj.globalt0
 
2503                      raise ValueError('Hybrid trajectory sequence time intervals'
 
2504                                       ' must be contiguous: ' + \
 
2505                                       str(traj.indepdomain[0]) + ' vs. ' + \
 
2506                                       str(time_range[1]-traj.globalt0)) 
2507                  time_range[1] += traj.indepdomain[1] 
2508                  time_partitions.append((traj.indepdomain, \
 
2509                                          traj.globalt0, \
 
2510                                          traj.checklevel)) 
2511          # Full time interval of the trajectory
 
2512          time_interval = Interval(indepvarname, DS_t_numtype, time_range,
 
2513                                   abseps=self._abseps) 
2514          # Add to trajectory dictionary, using hierarchical event names for
 
2515          # genEvents and genEventTimes (which are called directly by user methods
 
2516          # of Model)
 
2517          self.trajectories.update({trajname: \
 
2518                  HybridTrajectory(trajname, trajseq,
 
2519                                   timePartitions=time_partitions,
 
2520                                   timeInterval=time_interval,
 
2521                                   eventTimes=genEventTimes,
 
2522                                   events=genEvents,
 
2523                                   modelEventStructs=genEvStructs,
 
2524                                   modelNames=modelNames,
 
2525                                   FScompatibleNames=FScompatibleNames,
 
2526                                   FScompatibleNamesInv=FScompatibleNamesInv,
 
2527                                   abseps=self._abseps,
 
2528                                   globalt0=0, norm=self._normord)
 
2529                                    })

2530  
 


2531 -    def haveJacobian(self):


2532          """Returns True iff all objects in modelInfo have
 
2533          defined Jacobians.""" 
2534          result = True 
2535          for model in self.registry.values(): 
2536              result = result and model.haveJacobian() 
2537          return result

2538  
 


2539 -    def haveJacobian_pars(self):


2540          """Returns True iff all objects in modelInfo have
 
2541          defined Jacobians.""" 
2542          result = True 
2543          for model in self.registry.values(): 
2544              result = result and model.haveJacobian_pars() 
2545          return result

2546  
 


2547 -    def Rhs(self, dsName, t, xdict, pdict=None, asarray=False):


2548          """Direct access to a sub-model generator's Rhs function.
 
2549          Arguments:
 
2550  
 
2551            dsName   Name of a sub-model
 
2552            t        time (can use 0 for an autonomous system)
 
2553            xdict    state dictionary or Point.
 
2554            pdict    parameter dictionary or Point
 
2555                     (optional, default current parameters)
 
2556            asarray  (Bool, optional, default False) If true, will return an array
 
2557                     in state name alphabetical order, else a Point
 
2558          """ 
2559          try: 
2560              dsi = self.modelInfo[dsName]['dsi'] 
2561          except KeyError: 
2562              raise ValueError("No DS named %s was found"%dsName) 
2563          # don't need to convert pdict names to FS-compatible as they sort
 
2564          # the same
 
2565          if pdict is None: 
2566              pdict = self.pars 
2567          if asarray: 
2568              return dsi.Rhs(t, xdict, pdict, asarray=True) 
2569          else: 
2570              # get returns FS compatible names
 
2571              varnames = dsi.get('funcspec', xdict, t).vars 
2572              return Point({'coorddict': dict(zip(varnames,
 
2573                                  dsi.Rhs(t, xdict, pdict))),
 
2574                        'coordtype': float,
 
2575                        'norm': self._normord})

2576  
 


2577 -    def Jacobian(self, dsName, t, xdict, pdict=None, asarray=False):


2578          """Direct access to a sub-model generator's Jacobian function (if defined).
 
2579          Arguments:
 
2580  
 
2581            dsName   Name of a sub-model
 
2582            t        time (can use 0 for an autonomous system)
 
2583            xdict    state dictionary or Point.
 
2584            pdict    parameter dictionary or Point
 
2585                     (optional, default current parameters)
 
2586            asarray  (Bool, optional, default False) If true, will return an array
 
2587                     in state name alphabetical order, else a Point
 
2588          """ 
2589          try: 
2590              dsi = self.modelInfo[dsName]['dsi'] 
2591          except KeyError: 
2592              raise ValueError("No DS named %s was found"%dsName) 
2593          # don't need to convert pdict names to FS-compatible as they sort
 
2594          # the same
 
2595          if pdict is None: 
2596              pdict = self.pars 
2597          if dsi.haveJacobian(): 
2598              if asarray: 
2599                  return dsi.Jacobian(t, xdict, pdict, asarray=True) 
2600              else: 
2601                  varnames = dsi.get('funcspec', xdict, t).vars 
2602                  return Pointset({'coorddict': dict(zip(varnames,
 
2603                                                dsi.Jacobian(t, xdict, pdict))),
 
2604                               'coordtype': float,
 
2605                               'norm': self._normord}) 
2606          else: 
2607              raise PyDSTool_ExistError("Jacobian not defined")

2608  
 


2609 -    def JacobianP(self, dsName, t, xdict, pdict=None, asarray=False):


2610          """Direct access to a generator's JacobianP function (if defined).
 
2611          Arguments:
 
2612  
 
2613            dsName   Name of a sub-model
 
2614            t        time (can use 0 for an autonomous system)
 
2615            xdict    state dictionary or Point.
 
2616            pdict    parameter dictionary or Point
 
2617                     (optional, default current parameters)
 
2618            asarray  (Bool, optional, default False) If true, will return an array
 
2619                     in state name alphabetical order, else a Point
 
2620          """ 
2621          try: 
2622              dsi = self.modelInfo[dsName]['dsi'] 
2623          except KeyError: 
2624              raise ValueError("No DS named %s was found"%dsName) 
2625          if dsi.haveJacobian_pars(): 
2626              # don't need to convert pdict names to FS-compatible as they sort
 
2627              # the same
 
2628              if pdict is None: 
2629                  pdict = self.pars 
2630              if asarray: 
2631                  return dsi.JacobianP(t, xdict, pdict, asarray=True) 
2632              else: 
2633                  parnames = dsi.get('funcspec', xdict, t).pars 
2634                  return Pointset({'coorddict': dict(zip(parnames,
 
2635                                      dsi.JacobianP(t, xdict, pdict))),
 
2636                               'coordtype': float,
 
2637                               'norm': self._normord}) 
2638          else: 
2639              raise PyDSTool_ExistError("Jacobian w.r.t. pars not defined")

2640  
 


2641 -    def MassMatrix(self, dsName, t, xdict, pdict=None, asarray=False):


2642          """Direct access to a generator's MassMatrix function (if defined).
 
2643          Arguments:
 
2644  
 
2645            dsName   Name of a sub-model
 
2646            t        time (can use 0 for an autonomous system)
 
2647            xdict    state dictionary or Point.
 
2648            pdict    parameter dictionary or Point
 
2649                     (optional, default current parameters)
 
2650            asarray  (Bool, optional, default False) If true, will return an array
 
2651                     in state name alphabetical order, else a Point
 
2652          """ 
2653          try: 
2654              dsi = self.modelInfo[dsName]['dsi'] 
2655          except KeyError: 
2656              raise ValueError("No DS named %s was found"%dsName) 
2657          # don't need to convert pdict names to FS-compatible as they sort
 
2658          # the same
 
2659          if pdict is None: 
2660              pdict = self.pars 
2661          if asarray: 
2662              dsi.MassMatrix(t, xdict, pdict, asarray=True) 
2663          else: 
2664              varnames = dsi.get('funcspec', xdict, t).vars 
2665              return Point({'coorddict': dict(zip(varnames,
 
2666                              dsi.MassMatrix(t, xdict, pdict))),
 
2667                        'coordtype': float,
 
2668                        'norm': self._normord})

2669  
 


2670 -    def AuxVars(self, dsName, t, xdict, pdict=None, asarray=False):


2671          """Direct access to a generator's auxiliary variables
 
2672          definition (if defined).
 
2673  
 
2674          Arguments:
 
2675  
 
2676            dsName   Name of a sub-model
 
2677            t        time (can use 0 for an autonomous system)
 
2678            xdict    state dictionary or Point.
 
2679            pdict    parameter dictionary or Point
 
2680                     (optional, default current parameters)
 
2681            asarray  (Bool, optional, default False) If true, will return an array
 
2682                     in state name alphabetical order, else a Point
 
2683          """ 
2684          try: 
2685              dsi = self.modelInfo[dsName]['dsi'] 
2686          except KeyError: 
2687              raise ValueError("No DS named %s was found"%dsName) 
2688          # don't need to convert pdict names to FS-compatible as they sort
 
2689          # the same
 
2690          if pdict is None: 
2691              pdict = self.pars 
2692          if asarray: 
2693              return dsi.AuxVars(t, xdict, pdict, asarray=True) 
2694          else: 
2695              auxvarnames = dsi.get('funcspec', xdict, t).auxvars 
2696              return Point({'coorddict': dict(zip(auxvarnames,
 
2697                                  dsi.AuxVars(t, xdict, pdict))),
 
2698                        'coordtype': float,
 
2699                        'norm': self._normord})

2700  
 


2701 -    def compute(self, trajname, **kw):


2702          """Compute a hybrid trajectory and store it internally in the 'trajectories'
 
2703          attribute.
 
2704  
 
2705          Arguments (non-keyword):
 
2706            trajname   Name of trajectory to create (string)
 
2707  
 
2708          Arguments (keyword only, all optional):
 
2709            force      (Bool, default False) - force overwrite of any trajectory
 
2710                       stored in this object with the same name
 
2711            verboselevel  (int, default 0)
 
2712            ics        initial conditions dict or Point
 
2713            pars       parameters dict or Point
 
2714            tdata      time data (interval as sequence of 2 numeric values)
 
2715          """ 
2716          # initially expect to compute over a global time interval,
 
2717          # [t0_global, t1_global], which is truncated if it extends beyond
 
2718          # DS's independent variable domain to give [t0, t1].
 
2719          # DS tdata is then set to compute over relative time interval,
 
2720          # [0, t1-t0].
 
2721          tdata, t0_global, t1_global, force_overwrite = \
 
2722                  Model._prepareCompute(self, trajname, **kw) 
2723          # Set initial reason for an epoch to end to None to initiate
 
2724          # search for eligible Generator or Model for first epoch
 
2725          end_reasons = None 
2726          # check initial condition specification in icdict
 
2727          if self.icdict == {}: 
2728              raise PyDSTool_ExistError("No initial conditions specified") 
2729  
 
2730          xdict = {} 
2731          for xname, value in self.icdict.iteritems(): 
2732              if xname not in self.allvars: 
2733                  raise ValueError("Invalid variable name in initial "
 
2734                                     "conditions: " + xname) 
2735              xdict[xname] = ensurefloat(value) 
2736          # clean up self.icdict
 
2737          self.icdict = xdict.copy() 
2738  
 
2739          # initial values
 
2740          notDone = True  # finished computing trajectory segment? 
2741          t0 = t0_global 
2742          partition_num = 0 
2743          trajseq = [] 
2744          traj = None 
2745          ti_1 = None 
2746          modelNames = [] 
2747          epochEvents = [] 
2748          MI_prev = None 
2749          MI = None 
2750          swRules = None 
2751          # flag for re-use of a model from one hybrid segment to the next
 
2752          reused = False 
2753          # reset persistent storage of event times
 
2754          self.resetEventTimes() 
2755  
 
2756          # From t0 (if non-autonomous system), icdict, and switching rules
 
2757          # (self-consistency validity conditions), determine which model
 
2758          # applies for the initial portion of the trajectory
 
2759          while notDone: 
2760              # find appropriate model to compute trajectory segment
 
2761              # (reused flag indicates whether MI is the same as on previous loop
 
2762              # but is not used at the moment)
 
2763              MI, swRules, globalConRules, nextModelName, reused, \
 
2764                  epochStateMaps, notDone = self._findTrajInitiator(end_reasons,
 
2765                                                        partition_num, t0, xdict,
 
2766                                                        MI, swRules) 
2767              if not notDone: 
2768                  continue 
2769  
 
2770              # convenient shorthand
 
2771              model = MI.model 
2772  
 
2773              if partition_num > 0: 
2774                  ## map previous ds state
 
2775                  # to new ds state using epoch mapping
 
2776                  # and current ds's pars and external inputs
 
2777                  # (previous) traj is well defined from previous run
 
2778                  pars_copy = copy.copy(self.pars) 
2779                  self._applyStateMap(epochStateMaps, MI_prev, MI, traj, xdict, t0) 
2780                  ## if changed last MI's event delay, then change it back.
 
2781                  for event, delay in event_delay_record.items(): 
2782                      event.eventdelay = delay 
2783  
 
2784                  ## temporarily set terminal events from previous in this MI to have
 
2785                  # event_delay = event_interval because event finding code
 
2786                  # does not know that an event just occurred in previous MI and
 
2787                  # partition.
 
2788                  event_delay_record = {} 
2789                  dummy1, dummy2, targetMI = MI._get_initiator_cache(xdict, t0) 
2790                  evstruct = targetMI.get('eventstruct', xdict, t0) 
2791                  # record change for later resetting
 
2792                  for reason in end_reasons: 
2793                      # should only be one!
 
2794                      try: 
2795                          event = evstruct.events[reason] 
2796                      except KeyError: 
2797                          pass 
2798                      else: 
2799                          event_delay_record[event] = event.eventdelay 
2800                          event.eventdelay = event.eventinterval 
2801              else: 
2802                  # way to force partition 0 to definitely update any
 
2803                  # parameter-linked input t0's
 
2804                  pars_copy = {} 
2805                  event_delay_record = {} 
2806  
 
2807              # if external inputs used, make sure any parameter-bound
 
2808              # global t0 offsets are updated in case epoch state map changed them
 
2809              if self._inputt0_par_links != {} and pars_copy != self.pars: 
2810                  t0val_dict = {} 
2811                  for inp, val in self._inputt0_par_links.items(): 
2812                      t0val_dict[inp] = self.pars[val] 
2813                  model.set(inputs_t0=t0val_dict) 
2814  
 
2815              ## compute trajectory segment until an event or t1 is reached
 
2816              indepvar = MI.get('indepvariable', xdict, t0) 
2817              if t1_global > indepvar.indepdomain[1]+t0: 
2818                  if isfinite(indepvar.indepdomain[1]): 
2819                      if self.verboselevel > 0: 
2820                          print "Warning: end time was truncated to max size " + \
 
2821                                "of specified independent variable domain" 
2822                      t1 = indepvar.indepdomain[1]+t0 
2823                  else: 
2824                      t1 = t1_global 
2825              else: 
2826                  t1 = t1_global 
2827  
 
2828              # For Models that support setPars() and
 
2829              # compute() methods, set the global time
 
2830              # reference (in case it needs it), and verify that t=0 is valid
 
2831              # in the time domain
 
2832              ## next line redundant (and changed) by setup_pars['tdata'] below
 
2833  #            print "\nHybrid model %s:  set tdata=[0, %f] in %s (type %s)"%(self.name, t1-t0, MI.model.name, str((type(MI), type(MI.model))))
 
2834  #            print "  H model t0, t1 =", t0, t1
 
2835  #            print "  and globalt0 = ", t0
 
2836              MI.set('tdata', [0, t1-t0], xdict, t0) 
2837              MI.set('globalt0', t0, xdict, t0) 
2838  
 
2839              # add remaining pars for system
 
2840              setup_pars = {'ics': xdict, 'algparams': {}} 
2841              #if self._abseps is not None:
 
2842              #    setup_pars['abseps'] = self._abseps
 
2843              #    setup_pars['algparams'].update({'abseps': self._abseps})
 
2844              try: 
2845                  if MI.get('algparams', xdict, t0)['init_step'] > t1-t0: 
2846                      if self.verboselevel > 0: 
2847                          print "Warning: time step too large for remaining time"\
 
2848                              + " interval. Temporarily reducing time step to " \
 
2849                              + "1/10th of its previous value" 
2850                      setup_pars['algparams'].update({'init_step': (t1-t0)/10}) 
2851              except (AttributeError, KeyError, ValueError): 
2852                  # system does not support this integration parameter
 
2853                  pass 
2854              else: 
2855                  # we know there's compatibility to set verbose level
 
2856                  setup_pars['algparams']['verbose'] = self.verboselevel 
2857              model.set(**setup_pars) 
2858              # ensure that if reusing same model as previous segment, that
 
2859              # any high level events are not reset: especially for VODE and map system
 
2860              # (!!! unsure why not needed for Dopri & Radau)
 
2861              try: 
2862                  if modelNames[-1] == model.name: 
2863                      # reusing same model again
 
2864                      model._set_for_hybrid_DS(True) 
2865              except IndexError: 
2866                  # first hybrid partition, so doesn't apply
 
2867                  pass 
2868              model.diagnostics.clearWarnings() 
2869              model.diagnostics.clearErrors() 
2870  #            assert model._abseps == self._abseps
 
2871  
 
2872              #### Compute a trajectory segment (as a Hybrid Trajectory)
 
2873  #            print "DEBUG: Entering model %s at t0=%f, t1=%f"%(model.name, t0, t1)
 
2874              try: 
2875                  traj = MI.get_test_traj(force=True) 
2876              except PyDSTool_ValueError, e: 
2877                  print "\nError in Model:", model.name 
2878                  model.diagnostics.showWarnings() 
2879                  model.diagnostics.showErrors() 
2880                  print "Are the constituent generator absolute epsilon " 
2881                  print " tolerances too small? -- this abseps =", model._abseps 
2882                  raise 
2883              except KeyboardInterrupt: 
2884                  raise 
2885              except: 
2886                  print "\nError in Model:", model.name 
2887                  self.diagnostics.traceback = {} 
2888                  for k,v in model.diagnostics.traceback.iteritems(): 
2889                      if isinstance(v, dict): 
2890                          dentry = {} 
2891                          for vk, vv in v.iteritems(): 
2892                              dentry[traj._FScompatibleNamesInv(vk)] = vv 
2893                      else: 
2894                          dentry = v 
2895                      self.diagnostics.traceback[k] = dentry 
2896                  if self.diagnostics.traceback != {}: 
2897                      print "Traceback dictionary copied to model" 
2898                  raise 
2899              # ensure this is reset to False
 
2900              model._set_for_hybrid_DS(False) 
2901              if traj is None: 
2902                  raise ValueError("Model %s failed to create a trajectory"%model.name) 
2903              else: 
2904                  # rename from ModelInterface default name
 
2905                  model.renameTraj(MProject.ModelInterface._trajname,
 
2906                                   trajname+'_'+str(partition_num),
 
2907                                   force=force_overwrite) 
2908  #                if self._abseps is not None:
 
2909  #                    assert traj.indepdomain._abseps == self._abseps
 
2910  #                    for v in traj.depdomain.values():
 
2911  #                        assert v._abseps == self._abseps
 
2912                  if not isparameterized(traj): 
2913                      raise ValueError("Model " + model.name + " produced a " \
 
2914                                       + " non-parameterized Trajectory") 
2915                  time_interval = traj.indepdomain 
2916                  # ti_1 means time interval index 1 (i.e., the endpoint)
 
2917                  # this is a local time
 
2918                  ti_1 = time_interval[1] 
2919                  if self.verboselevel > 1: 
2920                      print "\nt0=", t0, "traj.globalt0=", traj.globalt0 
2921                      print "traj.indepdomain = ", traj.indepdomain.get() 
2922                      for vn, v in traj.variables.items(): 
2923                          print vn + ":", v.trajirange, v.indepdomain.get() 
2924                      print "Last point of traj %s was: "%traj.name, traj(ti_1) 
2925              # update original copy of the model with new event times during
 
2926              # this trajectory, for high level event detection to be able to check
 
2927              # eventinterval for terminal events between hybrid steps
 
2928              # ?????
 
2929              ##MI.eventstruct = copy.copy(model.eventstruct)
 
2930  
 
2931              # append record of the sequence of used generators
 
2932              modelNames.append(model.name) 
2933  
 
2934              #### Post-process trajectory segment:
 
2935              # look at warnings etc. for any terminating events that occurred
 
2936              if model.diagnostics.hasErrors() and self.verboselevel > 0: 
2937                  for e in model.diagnostics.errors: 
2938                      print 'Model ' + model.name + \
 
2939                                          ' in trajectory segment had errors' 
2940                      model.diagnostics.showErrors() 
2941              # default end reason is time (may be overwritten)
 
2942              end_reasons = ['time'] 
2943              # Default time interval after traj computation completed,
 
2944              # in case it was truncated by a terminal event
 
2945              # DEBUG
 
2946  #            print "Hybrid Traj %s t0, ti_1 ="%traj.name, traj.indepdomain[0], ti_1
 
2947              if model.diagnostics.hasWarnings(): 
2948                  if self.verboselevel >= 1: 
2949                      model.diagnostics.showWarnings() 
2950                  for w in model.diagnostics.warnings: 
2951                      if w[0] == Generator.W_TERMEVENT or \
 
2952                         w[0] == Generator.W_TERMSTATEBD: 
2953                          if self.verboselevel > 1: 
2954                              print "Time:", ti_1+t0 
2955                              print 'Model ' + model.name + \
 
2956                                  ' had a terminal event. Details (in local ' + \
 
2957                                  'system time) ...\n ' + str(w[1]) 
2958                          # w[1] always has t as first entry, and
 
2959                          # either a state variable name
 
2960                          # or a list of terminal event names as second.
 
2961                          if isinstance(w[1][1], list): 
2962                              if len(w[1][1]) > 1: 
2963                                  if self.verboselevel > 0: 
2964                                      print 'Warning: More than one terminal event found.' 
2965                                      print '  Consider altering event pars.' 
2966                              end_reasons = [w[1][1][ri] \
 
2967                                     for ri in range(len(w[1][1]))] 
2968                          else: 
2969                              end_reasons = [w[1][1]] 
2970                          #print "End reason: ", end_reasons[0]
 
2971                  self.diagnostics.update(model.diagnostics) 
2972  
 
2973              # Apply global consistency conditions, and truncate
 
2974              # trajectory to the last OK index in the independent variable
 
2975              # if any fail, for those conditions that do not apply to the
 
2976              # whole trajectory in an accept/reject fashion. If any fail
 
2977              # without specifying an index then the whole trajectory is
 
2978              # rejected.
 
2979              global_end_reasons = {} 
2980              global_end_ixs = [] 
2981              for globalDS in globalConRules: 
2982                  if globalDS(MI): 
2983                      global_end_reasons[Inf] = \
 
2984                              globalDS.conditions.collate_results('reasons',
 
2985                                                              merge_lists=True) 
2986                      global_end_ixs.append(Inf) 
2987                  else: 
2988                      # if global consistency fails then the features *must* provide
 
2989                      # the last OK position in the trajectory to truncate to,
 
2990                      # otherwise the whole trajectory FAILs.
 
2991                      try: 
2992                          final_ok_idx = globalDS.conditions._find_idx() 
2993                      except (AttributeError, RuntimeError): 
2994                          print "Trajectory creation failed..." 
2995                          globalDS.conditions.results.info() 
2996                          raise PyDSTool_ExistError("Global consistency checks failed for"
 
2997                                  " model interface %s for trajectory %s"%(str(globalDS),
 
2998                                                                       traj.name)) 
2999                      else: 
3000                          if final_ok_idx is None: 
3001                              final_ok_idx = Inf 
3002                          global_end_ixs.append(final_ok_idx) 
3003                          global_end_reasons[final_ok_idx] = \
 
3004                                  globalDS.conditions.collate_results('reasons',
 
3005                                                              merge_lists=True) 
3006              if len(global_end_reasons) > 0: 
3007                  smallest_ok_idx = min(global_end_ixs) 
3008                  # this idx may be Inf if globalDS passed all points OK
 
3009                  # - but it may have still changed (refined) the end reason
 
3010                  if isfinite(smallest_ok_idx): 
3011                      # truncate in place
 
3012                      traj.truncate_to_idx(smallest_ok_idx) 
3013                  # overwrite current end reason only if it's non-empty
 
3014                  new_reason = global_end_reasons[smallest_ok_idx] 
3015                  if new_reason != []: 
3016                      end_reasons = new_reason 
3017              if self.verboselevel > 1: 
3018                  print "End reason in %s was %s"%(self.name,str(end_reasons)) 
3019  
 
3020              # DEBUG
 
3021  #            print "\nTraj segment %i info:"%partition_num, traj.indepdomain.get(), traj.globalt0, type(traj)
 
3022  #            print "\n"
 
3023  
 
3024              #### Prepare for next partition & clean up
 
3025              partition_num += 1 
3026              if time_interval.atEndPoint(t1_global-t0, 'hi'): 
3027                  # we reached end of desired computation interval
 
3028                  notDone = False 
3029              if ti_1 > t1_global-t0: 
3030                  # We computed too far for the time interval
 
3031                  print "Warning: Segment time interval exceeds prescribed limits:" 
3032                  print " ... ", ti_1, " > ", t1_global-t0 
3033                  notDone = False 
3034              # last Model's time == t1 should hold after successful traj computation
 
3035              # otherwise t1 needs to be updated according to last terminating
 
3036              # event time point
 
3037              if ti_1 < t1-t0: 
3038                  if end_reasons[0] not in swRules: 
3039                      # there's prescribed time left to compute but no
 
3040                      # eligible DS to transfer control to
 
3041                      print 'Trajectory calculation for Model `'+model.name+'` ' \
 
3042                            +'terminated without a DS specified in the ' \
 
3043                            +'switching rules from which to continue.' 
3044                      print 'Perhaps a variable went out of bounds:' 
3045                      model.diagnostics.showWarnings() 
3046                      print 'Last reasons for stopping were: ', end_reasons 
3047                      print "Trajectory calculated: " 
3048                      dt_sample = (ti_1-time_interval[0])/10. 
3049                      print traj.sample(dt=dt_sample) 
3050                      notDone = False 
3051                      raise PyDSTool_ValueError("Premature termination of "
 
3052                                                "trajectory calculation") 
3053                  elif swRules[end_reasons[0]][0] == 'terminate': 
3054                      if self.verboselevel > 0: 
3055                          print 'Trajectory calculation for Model `'+model.name+'` ' \
 
3056                                +'terminated.' 
3057                      notDone = False 
3058                      t1 = ti_1+t0 
3059                  else: 
3060                      t1 = ti_1+t0 
3061              # update xdict with final point in traj
 
3062              self._prepareICs(xdict, traj, MI, t0, ti_1) 
3063              # store trajectory segment in the sequence
 
3064              trajseq.append(traj) 
3065              # before moving on, update t0 for next epoch / regime
 
3066              t0 = t1 
3067              try: 
3068                  epochEvents.append(traj.getEvents()) 
3069              except AttributeError: 
3070                  # no eventstruct present
 
3071                  epochEvents.append({}) 
3072              MI_prev = MI 
3073          # end while loop
 
3074  
 
3075          # Take a copy of the events as they were at the time that the
 
3076          # trajectory was computed, for future reference
 
3077          self._addTraj(trajname, trajseq,
 
3078                        epochEvents, modelNames, force_overwrite) 
3079          # Record defining data for this trajectory (especially useful for interfaces)
 
3080          self.trajectory_defining_args[trajname] = args(ics=copy.copy(self.icdict),
 
3081                                                pars=copy.copy(self.pars),
 
3082                                                tdata=copy.copy(tdata))

3083  
 


3084 -    def _validateRegistry(self, obsvars, intvars):


3085          """Validate Model's modelInfo attribute.
 
3086          Now that a HybridModel is invariably built using ModelConstructor,
 
3087          which already has validation built in, and also has more complex ways
 
3088          of construction, this method is defunct.""" 
3089          pass

3090  
 
3091  ##        # ensure that modelInfo consists of more than one ModelInterface object
 
3092  ##        # unless it's a single model punctuated by discrete events
 
3093  ##        if len(self.modelInfo) == 1:
 
3094  ##            infodict = self.modelInfo.values()[0]
 
3095  ##            swmapping = infodict['swRules']
 
3096  ##            if all([outcome[0] == 'terminate' \
 
3097  ##                   for (reason, outcome) in swmapping.iteritems()]):
 
3098  ##                # then all point to 'terminate' and user needed a non-hybrid
 
3099  ##                # model class, otherwise has discrete event mappings
 
3100  ##                # that make the model technically "hybrid"
 
3101  ##                raise AssertionError("Use a non-hybrid Model class")
 
3102  ##        for infodict in self.modelInfo.values():
 
3103  ##            if not isinstance(infodict['dsi'], MProject.ModelInterface):
 
3104  ##                raise TypeError("Must provide ModelInterface objects")
 
3105  ##        allDSnames = self.modelInfo.keys()
 
3106  ###        print "\n_validateRegistry: ", obsvars, intvars
 
3107  ##        for modelName, infodict in self.modelInfo.iteritems():
 
3108  ##            # dynamical system for trajectory
 
3109  ##            miface = infodict['dsi']
 
3110  ##            # dict of reason -> (ds_name, epmapping) pairs
 
3111  ##            swmapping = infodict['swRules']
 
3112  ##            allvarnames = miface.model.query('variables')
 
3113  ###            print "Model %s had vars "%modelName, allvarnames
 
3114  ##            special_reasons = ['time'] + allvarnames
 
3115  ##            assert miface.model.name == modelName, ('DS`s name does not '
 
3116  ##                                'correspond to the key in the modelInfo dict')
 
3117  ##            assert miface.model.name in allDSnames, ('DS`s name not in list '
 
3118  ##                                        'of all available DS names!')
 
3119  ##            assert len(intersect(obsvars, allvarnames)) ==\
 
3120  ##                len(obsvars), 'DS `'+miface.model.name+'` did not contain ' \
 
3121  ##                                      + ' required observable variables'
 
3122  ##            assert len(intersect(intvars, allvarnames)) ==\
 
3123  ##                len(intvars), 'DS '+miface.model.name+' did not contain ' \
 
3124  ##                                      + ' required internal variables'
 
3125  ##            try:
 
3126  ##                allEndReasonNames = miface.model.query('events').keys() \
 
3127  ##                                      + special_reasons
 
3128  ##            except AttributeError:
 
3129  ##                allEndReasonNames = special_reasons
 
3130  ##            seenReasons = []
 
3131  ##            # can't assert this when non-event based reasons are used
 
3132  ##            # (e.g. for DSSRT post-processing which creates the reason based on
 
3133  ##            # generic events)
 
3134  ###            assert len(swmapping) == len(allEndReasonNames), ('Incorrect number'
 
3135  ###                                            ' of map pairs given in argument')
 
3136  ##            for (reason, outcome) in swmapping.iteritems():
 
3137  ##                targetName = outcome[0]
 
3138  ##                epmapping = outcome[1]
 
3139  ##                # no checks are made on epmapping here
 
3140  ##                assert reason not in seenReasons, ('reasons cannot appear more'
 
3141  ##                                            ' than once in map domain')
 
3142  ##                seenReasons.append(reason)
 
3143  ##                assert reason in allEndReasonNames, ('name `'+reason+'` in map '
 
3144  ##                                                    'domain is invalid')
 
3145  ##                if targetName != 'terminate':
 
3146  ##                    assert targetName in allDSnames, ('name `'+targetName+\
 
3147  ##                                            '` in map range is invalid')
 
3148  
 
3149  
 
3150  
 


3151 -    def _validateParameters(self):


3152          pars_found = {} 
3153          for infodict in self.modelInfo.values(): 
3154              try: 
3155                  pars_recognized = intersect(pars_found.keys(),
 
3156                                          infodict['dsi'].pars) 
3157              except AttributeError: 
3158                  # gen doesn't have any pars
 
3159                  continue  # for loop 
3160              pars_unknown = remain(infodict['dsi'].pars, pars_found.keys()) 
3161              for parname in pars_recognized: 
3162                  if infodict['dsi'].pars[parname] != pars_found[parname]: 
3163                      print "Inconsistent parameter values between model objects inside model" 
3164                      print "probably means that they were changed during trajectory computation" 
3165                      print "at discrete events: you should reset the pars when calling compute()" 
3166                      raise ValueError("Inconsistency between parameter values"
 
3167                       " of same name ('%s') in different constiutent Generator"%parname +\
 
3168                       " objects: reset during call to compute") 
3169              new_vals = dict(zip(pars_unknown, [infodict['dsi'].pars[p] \
 
3170                                                 for p in pars_unknown])) 
3171              pars_found.update(new_vals)

3172  
 
3173  
 


3174 -    def _infostr(self, verbose=1):


3175          if verbose > 0: 
3176              outputStr = 'Hybrid Model '+self.name+" containing components:" 
3177              outputStr += "Observable variables: " + ",".join(self.obsvars) 
3178              outputStr += "Internal variables: " + ",".join(self.intvars) 
3179              outputStr += "Auxiliary variables: " + ",".join(self.auxvars) 
3180              for name, infodict in self.modelInfo.iteritems(): 
3181                  outputStr += "\n--- Sub-model: "+name 
3182                  outputStr += "\n  "+infodict['dsi'].model._infostr(verbose-1) 
3183          else: 
3184              outputStr = 'Hybrid Model '+self.name 
3185          return outputStr

3186  
 
3187  
 
3188  # -------------------------------------------------------------------------
 
3189  #### Private functions
 
3190  
 


3191 -def getAuxVars(dsi, t, icdict, pardict):


3192      """Return auxiliary variable values evaluated at t, icdict,
 
3193      pardict.
 
3194      """ 
3195      # prepare auxiliary variables eval'd at i.c.
 
3196      icdict_local = copy.copy(icdict) 
3197      # to restore these to their former values after domain tests are done,
 
3198      # (in case events refer to auxiliary function initcond()
 
3199      old_icdict = copy.copy(dsi.model.icdict) 
3200      # update icdict with auxiliary variable vals eval'd at t, for
 
3201      # use in event function calls for domain test.
 
3202      dsi.model.icdict.update(icdict_local) 
3203      icdict_local['t'] = t 
3204      fspec = dsi.get('funcspec', icdict, t) 
3205      if fspec.auxvars == []: 
3206          auxdict = {} 
3207      else: 
3208          try: 
3209              auxdict = dict(zip(fspec.auxvars,
 
3210                      apply(dsi.get('AuxVars', icdict, t),
 
3211                                          (t, icdict_local, pardict)) )) 
3212          except (ValueError, TypeError): 
3213              # no auxiliary variables for this model
 
3214              # e.g. ExplicitFnGen has its auxvars defined through the
 
3215              # main variables
 
3216              auxdict = {} 
3217      # restore model's original initial conditions
 
3218      dsi.model.icdict = old_icdict 
3219      return auxdict

3220  
 
3221  
 


3222 -def findTrajInitiator(modelInfo, t, vardict, pardict, intvars,
 
3223                           verboselevel=0):


3224      """Find eligible Model to begin computation of trajectory.
 
3225      Cannot depend on any internal variables (e.g. those not common to
 
3226      all sub-models).
 
3227      """ 
3228      eligibleMI = [] 
3229      if len(modelInfo) == 1: 
3230          return modelInfo.values()[0] 
3231      outcome = {} 
3232      for infodict in modelInfo.values(): 
3233          MI = infodict['dsi'] 
3234          MI_vars = MI.query('vars') 
3235          xdict = filteredDict(vardict, MI_vars) 
3236          dxdt_zeros = dict(zip(xdict.keys(),[0.]*len(xdict))) 
3237          domtests = infodict['domainTests'] 
3238          globalConRules = infodict['globalConRules'] 
3239          model = MI.model 
3240          outcome[model.name] = {} 
3241          t_test = MI.get('indepvariable', xdict, t).indepdomain.contains(t) \
 
3242                 is not notcontained 
3243          fs = MI.get('funcspec', xdict, t) 
3244          allvars = fs.vars + fs.auxvars 
3245          # if external inputs then include initial value of those in pardict
 
3246          dsinps = MI.get('inputs', xdict, t) 
3247          if dsinps != {}: 
3248              try: 
3249                  input_ics = [dsinps[in_name](t) for in_name in dsinps.keys()] 
3250              except KeyboardInterrupt: 
3251                  raise 
3252              except: 
3253                  raise RuntimeError("Error in evaluating inputs for generator at"
 
3254                                     " initial condition") 
3255              pardict.update(dict(zip(dsinps.keys(), input_ics))) 
3256          icdict = copy.copy(xdict) 
3257          # don't overwrite any aux vars that are treated as regular variables
 
3258          # at this level, but fetch other aux vars and external inputs
 
3259          icdict.update(filteredDict(getAuxVars(MI, t, icdict, pardict),
 
3260                                     icdict.keys(), neg=True)) 
3261          # override icdict with any finite-valued generator
 
3262          # initial condition (deliberately preset in generator definition)
 
3263          # for non-internal variables, and any auxiliary variables that aren't
 
3264          # already defined in icdict from above call to getAuxVars (must do this
 
3265          # to prevent NaNs being passed to numeric_to_traj.
 
3266          for xname in allvars: 
3267              if xname not in xdict or xname in intersect(intvars, xdict): 
3268                  if xname in xdict and xname in intvars: 
3269                      # ignore
 
3270                      continue 
3271                  if xname in fs.auxvars and xname in icdict and isfinite(icdict[xname]): 
3272                      continue 
3273                  try: 
3274                      if isfinite(model.icdict[xname]): 
3275                          icdict[xname] = model.icdict[xname] 
3276                  except KeyError: 
3277                      # ic (for internal variable) not defined for this
 
3278                      # generator, so ignore
 
3279                      pass 
3280          # set initial conditions of MI in case auxiliary function initcond
 
3281          # is called during Rhs call
 
3282          model.set(ics=icdict) 
3283          x_test = True   # initial value 
3284          try: 
3285              # derivatives of variables are just Rhs values for ODE
 
3286              dxdt = dict(MI.Rhs(t, icdict, pardict)) 
3287          except AttributeError: 
3288              # no Rhs for the underlying generator
 
3289              dxdt = dxdt_zeros 
3290          else: 
3291              for vname, val in dxdt.items(): 
3292                  # hack to fix NaNs showing up for VODE Rhs function
 
3293                  # but very large finite values come back from Dopri/Radau
 
3294                  # and Nan's mess up call to numeric_to_traj below
 
3295                  if not isfinite(val): 
3296                      dxdt[vname] = 1e308 
3297          for aname in fs.auxvars: 
3298              # set derivatives for remaining auxvars to dummy value
 
3299              dxdt[aname] = 0 
3300  #        print "Temp in findTrajInitiator for %s: x, dxdt ="%MI.model.name
 
3301  #        print icdict
 
3302  #        print dxdt
 
3303          for xname, x in icdict.iteritems(): 
3304              # don't count time 't' or any internal variables
 
3305              if xname == 't' or xname in intvars or xname not in domtests: 
3306                  # 't' is a special value inserted into icdict, for use
 
3307                  # in auxiliary variable evaluation at initial time, but not
 
3308                  # a regular variable so don't do domain check here.
 
3309                  continue 
3310              xtraj = numeric_to_traj([[x], [dxdt[xname]]], 'test', [xname, 'D_'+xname], t) 
3311  #            print "Dom test for %s in %s"%(xname, MI.model.name)
 
3312  #            print "  discrete? ", domtests[xname].isdiscrete
 
3313              newtest = domtests[xname](xtraj) 
3314              if verboselevel >=2: 
3315                  print "\nstate dom test for '%s' @ value %f:"%(xname, icdict[xname]) 
3316                  print "depdomain is ", MI.get('variables', xdict, t)[xname].depdomain.get() 
3317                  print "-> test result is ", newtest 
3318              x_test = x_test and newtest 
3319          g_test = True  # initial value 
3320          xdict = filteredDict(icdict, intvars, neg=True) 
3321          MI.test_traj = numeric_to_traj(array([xdict.values()]).T, 'ic_trajpt',
 
3322                                         xdict.keys(), t) 
3323          # ensure that events are cleared in case global con rules in a MI
 
3324          # check for events (for when used after trajectories computed)
 
3325          # don't use MI.get('diagnostics', xdict, t) as that will return a copy
 
3326          # so that clearWarnings will not propagate to underlying sub-model
 
3327          xdict, t, I = MI._get_initiator_cache(xdict, t) 
3328          while True: 
3329              # descend into sub-models until leaf found
 
3330              if isinstance(I, MProject.GeneratorInterface): 
3331                  I.model.diagnostics.clearWarnings() 
3332                  break 
3333              else: 
3334                  xdict, t, I = I._get_initiator_cache(xdict, t) 
3335  
 
3336          # !!! Currently assumes globalConRules is a list, not a Context
 
3337          # (should be upgraded to that)
 
3338          for gc in globalConRules: 
3339              try: 
3340                  globtest = gc(MI) 
3341              except KeyboardInterrupt: 
3342                  raise 
3343              except: 
3344                  globtest = False 
3345              else: 
3346                  if verboselevel >= 2: 
3347                      print "\nglobal con test for '%s' @ value %f:"%(str(gc), icdict[xname]) 
3348                      print "-> test result is ", globtest 
3349                  g_test = g_test and globtest 
3350          if verboselevel >= 1: 
3351              print "\nModel '%s' tests..."%model.name 
3352              print " ...for initial time: " + str(t_test) 
3353              print " ...for initial state: " + str(x_test) 
3354              print " ...for global conditions: " + str(g_test) 
3355          outcome[model.name]['time dom. test'] = t_test 
3356          outcome[model.name]['state dom. test'] = x_test 
3357          outcome[model.name]['global con. test'] = g_test 
3358          if t_test and x_test and g_test: 
3359              # this t, xdict is a valid initial state for this Generator
 
3360              eligibleMI.append(infodict) 
3361      if eligibleMI == []: 
3362          info(outcome, "\nOutcomes for eligibility tests, by model") 
3363          raise PyDSTool_ValueError('No eligible models from'
 
3364                             ' which to begin trajectory computation') 
3365      if len(eligibleMI) > 1: 
3366          info(outcome, "\nOutcomes for eligibility tests, by model") 
3367          raise PyDSTool_ValueError('Too many eligible models'
 
3368                             ' to start trajectory computation') 
3369      # only remaining possibility is a single eligible model
 
3370      return eligibleMI[0]

3371
```

  


| Home | Trees | Indices | Help | PyDSTool |
| --- | --- | --- | --- | --- |

|  |  |
| --- | --- |
| Generated by Epydoc 3.0.1 on Fri May 4 15:24:24 2012 | http://epydoc.sourceforge.net |
